# The mitochondrial tRNA-derived fragment, mt-tRF-Leu^TAA^, couples mitochondrial metabolism to insulin secretion

**DOI:** 10.1016/j.molmet.2024.101955

**Published:** 2024-05-03

**Authors:** Cecile Jacovetti, Chris Donnelly, Véronique Menoud, Mara Suleiman, Cristina Cosentino, Jonathan Sobel, Kejing Wu, Karim Bouzakri, Piero Marchetti, Claudiane Guay, Bengt Kayser, Romano Regazzi

**Affiliations:** 1 Department of Fundamental Neurosciences, University of Lausanne, Lausanne, Switzerland; 2 Institute of Sport Sciences, University of Lausanne, Lausanne, Switzerland; 3 Department of Clinical and Experimental Medicine, Diabetes Unit, University of Pisa, Pisa, Italy; 4 UMR DIATHEC, EA 7294, Centre Européen d’Etude du Diabète, Université de Strasbourg, Fédération de Médecine Translationnelle de Strasbourg, Strasbourg, France; 5 Department of Biomedical Sciences, University of Lausanne, Lausanne, Switzerland

**Keywords:** Insulin secretion, Mitochondrial OXPHOS, Mitochondrial tRNA-derived fragments

## Abstract

**Objective:**

The contribution of the mitochondrial electron transfer system to insulin secretion involves more than just energy provision. We identified a small RNA fragment (mt-tRF-Leu^TAA^) derived from the cleavage of a mitochondrially-encoded tRNA that is conserved between mice and humans. The role of mitochondrially-encoded tRNA-derived fragments remains unknown. This study aimed to characterize the impact of mt-tRF-Leu^TAA^, on mitochondrial metabolism and pancreatic islet functions.

**Methods:**

We used antisense oligonucleotides to reduce mt-tRF-Leu^TAA^ levels in primary rat and human islet cells, as well as in insulin-secreting cell lines. We performed a joint transcriptome and proteome analysis upon mt-tRF-Leu^TAA^ inhibition. Additionally, we employed pull-down assays followed by mass spectrometry to identify direct interactors of the fragment. Finally, we characterized the impact of mt-tRF-Leu^TAA^ silencing on the coupling between mitochondrial metabolism and insulin secretion using high-resolution respirometry and insulin secretion assays.

**Results:**

Our study unveils a modulation of mt-tRF-Leu^TAA^ levels in pancreatic islets in different Type 2 diabetes models and in response to changes in nutritional status. The level of the fragment is finely tuned by the mechanistic target of rapamycin complex 1. Located within mitochondria, mt-tRF-Leu^TAA^ interacts with core subunits and assembly factors of respiratory complexes of the electron transfer system. Silencing of mt-tRF-Leu^TAA^ in islet cells limits the inner mitochondrial membrane potential and impairs mitochondrial oxidative phosphorylation, predominantly by affecting the Succinate (via Complex II)-linked electron transfer pathway. Lowering mt-tRF-Leu^TAA^ impairs insulin secretion of rat and human pancreatic β-cells.

**Conclusions:**

Our findings indicate that mt-tRF-Leu^TAA^ interacts with electron transfer system complexes and is a pivotal regulator of mitochondrial oxidative phosphorylation and its coupling to insulin secretion.

## Highlights

• Functional impact and mechanistic signaling of mitochondrially-encoded tRNA-derived fragments are currently unknown.
• The level of the mitochondrially-encoded tRNA mt-tRF-LeuTAA in pancreatic islets varies with the nutritional status.
• The mt-tRF-LeuTAA fragment interacts with electron transfer system complexes.
• mt-tRF-LeuTAA regulates mitochondrial respiration and insulin release in rat and human β-cells.
• mt-tRF-LeuTAA silencing leads to impaired metabolism-insulin secretion coupling, a hallmark of diabetes.

## 1 Introduction

Insulin is a vital hormone regulating glucose and energy balance. The secretion of this hormone from pancreatic β-cells is triggered by a rise in blood glucose levels and is coupled to the processing of the carbohydrate through the glycolytic pathway and mitochondrial oxidative phosphorylation (OXPHOS) [1]. In mitochondria, the protonmotive force, composed of a chemical potential difference ΔpH and an electrical potential difference Δ*Ψ*_mt_, generated by the oxidation of reduced glucose, is used to drive the phosphorylation of ADP to ATP [2]. The consequent rise in the ATP/ADP ratio is coupled to insulin granule exocytosis via the closure of ATP-sensitive K^+^ channels, plasma membrane depolarization and calcium influx through voltage-gated Ca^2+^ channels [1,3].

Once secreted in the blood, insulin suppresses hepatic glucose production and facilitates glucose uptake by muscle and adipose tissue, thereby lowering blood glucose levels [4]. Prolonged nutritional excess in genetically and epigenetically susceptible individuals can lead to obesity-related type 2 diabetes (T2D), featuring hyperglycemia, disrupted lipid metabolism, and ATP depletion due to mitochondrial uncoupling [5,6]. Inadequate nutrition in early-life, stemming from maternal over- or undernutrition, can lead to altered fetal or neonatal β-cell programming and to increased T2D susceptibility [7].

An unhealthy diet in individuals suffering from obesity-associated diabetes, has been shown to induce epigenetic changes in pancreatic islets [8]. These changes include DNA methylation, histone modifications, and altered non-coding RNA-mediated processes [8]. Recently, high-throughput RNA sequencing unveiled the presence of a large number of small RNA molecules resulting from the cleavage of precursor or mature tRNAs [9]. The human genome contains 264 nuclear-encoded and 22 mitochondrially-encoded tRNAs (mt-tRNAs), which display cell- and tissue-specific expression [10]. tRNAs undergo extensive post-transcriptional modifications in response to environmental cues such as nutritional status, oxidative stress, hypoxia and hypothermia [10,11]. These changes affect tRNA stability, codon-anticodon recognition, and regulate the generation of tRNA-derived fragments (tRFs) – a new class of small non-coding RNAs [12]. Indeed, these modifications can either attract or repel endoribonucleases, influencing the subsequent processing and cleavage of the tRNAs [11]. tRFs can originate from nuclear- or mitochondrially-encoded tRNAs, while it is not clear whether the latter undergo cleavage by imported nuclear- or mitochondrially-encoded endoribonucleases [13,14]. tRFs have been proposed to participate in the regulation of protein translation, protein and RNA stability, RNA silencing, and cell survival [11,12]. Given that less than 5% of the total tRNA pool is cleaved, the effects of the tRFs are not the result of tRNA depletion [15,16]. Mutations in mt-tRNAs and in tRNA-modifying enzymes can cause tRNA hypomodifications, leading to altered tRNA fragmentation and promoting the development of a large spectrum of metabolic diseases, including diabetes [17]. However, the mechanistic link between changes in mt-tRFs and such metabolic diseases is currently lacking.

This study focused on a conserved mitochondrial fragment called mt-tRF-Leu^TAA^, derived from a tRNA encoded by the mitochondrial genome. We singled out this fragment after screening a range of mt-tRFs showing changes in islets of diabetes-prone rodents. We found that mt-tRF-Leu^TAA^ levels fluctuate in response to nutritional status and to the modulation of the mTORC1 pathway, in pancreatic islets of various animal models of metabolic disturbances. Silencing of mt-tRF-Leu^TAA^ limits Δ*Ψ*_mt_, decreases mitochondrial OXPHOS capacity and reduces insulin secretion from pancreatic β-cells. We provide evidence that the effects of mt-tRF-Leu^TAA^ may stem from its interaction with complexes of the electron transfer system (ETS) and other mitochondrial regulators.

## 2 Results

### 2.1 The level of a mitochondrially-encoded tRNA-derived fragment is reduced in pancreatic islets of diabetes-susceptible rodents

**Figure 1:**
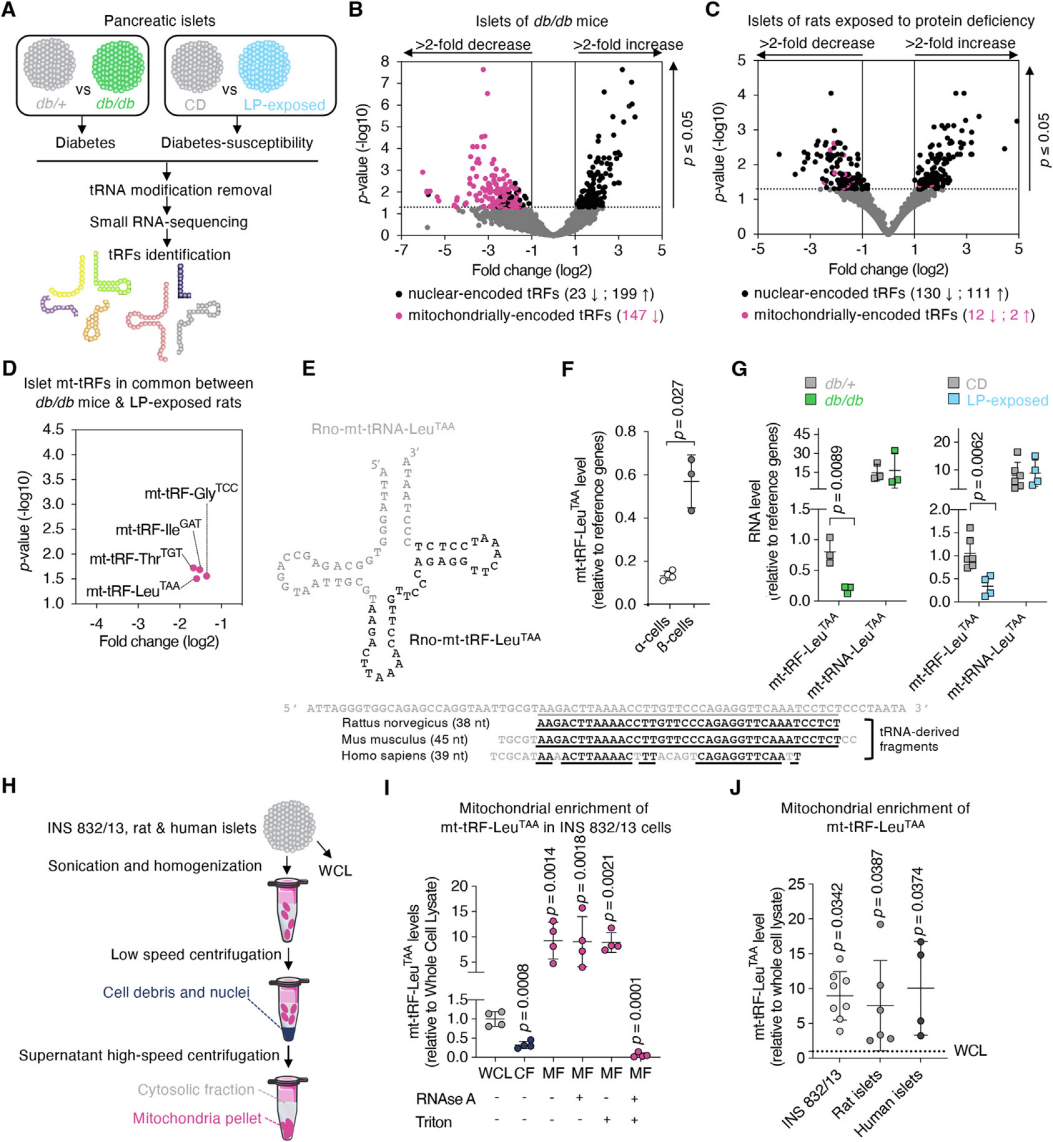
**Pancreatic islets of diabetes-susceptible rodents exhibit a reduction in a mitochondrially-encoded tRNA-derived fragment.** A. Experimental design to uncover the expression profile of tRNA-derived fragments (tRFs) in pancreatic islets of rodents prone to develop diabetes. *N* = 3 db*/+* heterozygote mice used as “control” vs *N* = 3 pre-diabetic 16-wk-old *db/db* mice. *N* = 5 22-day-old F1 progeny of control chow diet (CD)-fed rats vs *N* = 4 22-day-old F1 progeny of low-protein diet (LP)-fed rats. B. Volcano plot showing the 369 tRFs significantly changed amongst the 3858 tRFs detected in pancreatic islets of 16 wk-old *db/db* mice. 2 fold change cutoff, *p* ≤ 0.05. C. Volcano plot for the 255 tRFs significantly changed amongst the 6164 tRFs detected in pancreatic islets of 22 day-old LP-exposed rats. 2 fold change cutoff, *p* ≤ 0.05. D. Zoom in of the volcano plots for 4 of the tRFs that display significant changes (all 4 are more than 2 fold down-regulated) in islets of *db/db* mice and are also modified in islets of LP-exposed rats. E. Schematic representation of the Rattus norvegivus ortholog of the mitochondrially-encoded tRNA named mt-tRNA-Leu^TAA^ and of its cleaved fragment at the internal region, mt-tRF-Leu^TAA^. Sequence alignments of mt-tRF-Leu^TAA^ orthologs in rat, mouse and human. Underlined are the sequences conserved between rat, mouse and human. F. Measurement of the level of mt-tRF-Leu^TAA^ in rat FAC-sorted α- and β-cell fractions by real-time PCR. For relative comparison of tRF levels, see details in Methods and protocol section 5.4. Circles show individual values while error bars indicate SD, *N* = 3 to 4, unpaired Student's *t*-test, *p* ≤ 0.05. G. qRT-PCR to assess the levels of the mt-tRF-Leu^TAA^ fragment and of its host tRNA mt-tRNA-Leu^TAA^ in pancreatic islets of *db/db* vs *db/+* mice and of LP-exposed vs CD-exposed rats. For relative tRNA level comparison between samples, see details in Material and methods section 5.7. Squares represent individual values while error bars indicate SD, *N* = 3 to 6, unpaired Student's *t*-tests, *p* ≤ 0.05. H. Schematic representation of the protocol used to prepare whole cell lysates (WCL), cytosolic (CF) and mitochondrial fraction (MF) fractions from INS 832/13 rat insulinoma cell line, Wistar rat islets (RI) and human islets (HI). I. Relative levels of mt-tRF-Leu^TAA^ in CF and MF relative to WCL. Mitochondria were treated with RNAse A to remove contaminating RNAs surrounding the mitochondria. Triton was added to the MF to disrupt the mitochondrial membranes. The combination of Triton and RNAse A treatments depleted all RNAs present in the mitochondrial membranes, in the intermembrane space as well as in the mitochondrial matrix. Data represent fold changes relative to whole cell lysate, *N* = 4 independent experiments ±SD, one-way ANOVA with a Dunnett post-hoc test, *p* ≤ 0.05. J. Enrichment of mt-tRF-Leu^TAA^ in MF obtained from INS 832/13 rat insulinoma cell line, Wistar rat islets (RI) and human islets (HI) relative to WCL. Data are means of 4–8 independent experiments ±SD, one-way ANOVA followed by a Dunnett post-hoc test, *p* ≤ 0.05.

To evaluate the potential contribution of tRFs in the pathogenesis and progression of T2D, we determined the changes in the islet tRF profile occurring under diabetes and conditions predisposing to this metabolic disease. To this end, we analyzed by small-RNA sequencing the levels of the tRFs present in the islets of diabetic *db/db* mice (Figure 1A). These mice carry a mutation in the leptin receptor and, at 15–16 weeks of age, are hyperphagic, obese and exhibit fasting hyperglycaemia (Fig. S1A) [18]. We annotated 3858 tRFs present in the islets of *db/db* mice, 342 of which displaying significant changes (≥2-fold; adjusted *p*-value ≤0.05) compared to heterozygous (*db/+*) controls (GEO accession GSE239786) (Figure 1B). Among them, 199 tRFs were found to increase and 170 to decrease in the islets of diabetic animals. The vast majority (147/170) of the tRFs showing reduced levels originate from the cleavage of tRNAs encoded by the mitochondrial genome (mt-tRFs). These findings cannot be explained by an overall drop in mitochondrial transcription. In fact, the expression of mitochondrial ribosomal RNAs as well as the mitochondrial DNA (mt-DNA) content were not altered in the islets of *db/db* mice (Fig. S1B).

The so-called LP-exposed rat model consists of 22-day-old rats whose mothers have been subjected to a low protein (LP) diet during gestation and lactation (Figures 1A and S1C). Postnatally, these animals display impaired pancreatic development with reduced β-cell mass, defective glucose-stimulated insulin secretion (GSIS) and improved insulin sensitivity which transitions to insulin resistance (IR) and glucose intolerance in adulthood [19,20]. Among the 6164 tRFs detected in the islets of these newborn rats, 113 tRFs increased and 142 tRFs decreased in response to protein deficiency (GEO accession GSE239981) (Figure 1C). Both, *db/db* mice and LP-exposed rats, exhibit impaired insulin secretion and disturbances in mitochondrial oxidative capacities [18,21,22]. To unravel the potential impact of tRFs on diabetes pathogenesis, we focused on mt-tRFs displaying alterations in these two animal models. Four mt-tRFs were reduced both in the islets of *db/db* mice and in LP-exposed rats compared to their respective controls (Figure 1D). Of these, mt-tRF-Gly^TCC^ and mt-tRF-Thr^TGT^ exhibited expression levels nearing the detection limit while the mt-tRF-Ile^GAT^ includes 52 nucleotides. We currently lack tools to specifically reduce the level of such a long fragment without affecting the whole tRNA (Fig. S1D; see section 5.13). Thus, we focused on mt-tRF-Leu^TAA^ (Figure 1E), which was abundant in islet cells and was consistently down-regulated in both diabetes prone animal models.

The mitochondrial genome exhibits high conservation across mammals. This allowed us to identify mouse and human orthologs of rat mt-tRF-Leu^TAA^ (Figure 1E) [23]. Fluorescence-activated cell sorting (FACS) of rat islet cells revealed that mt-tRF-Leu^TAA^ was predominantly found in the β-cell fraction compared to α-cell fraction (Figure 1F). These findings suggest that changes in mt-tRF-Leu^TAA^ level measured in islets of diabetes prone animal models occurred mainly in insulin-secreting cells. Real-time PCR measurements showed that the fragment is 10–20 times less abundant compared to its host tRNA. In contrast to the fragment, the level of the whole mt-tRNA-Leu^TAA^ was unaffected in the islets of *db/db* mice and LP-exposed rats (Figure 1G).

tRNAs undergo post-transcriptional modifications which affect their stability, their function, their fragmentation and potentially their detection. Even though mitochondrially-encoded tRNAs are generally less modified than those encoded by the nuclear genome [10], we compared the amplification via qRT-PCR of mt-tRF-Leu^TAA^ in samples in which the most common modifications were removed. We found that the detection of the fragment was not affected by the removal of the modifications, suggesting that the fragment is unlikely to contain methylations or acetylations (Fig. S1E). Subcellular fractionation of insulin-secreting INS 832/13 cells (Figure 1H) revealed that mt-tRF-Leu^TAA^ was approximately 10-fold more abundant in mitochondrial fractions (MF) compared to whole cell lysates (WCL) (Figure 1I). Similar enrichments were observed in MFs of rat (RI) and human islets (HI) (Figure 1J). Treatment of MFs with RNAse A, which removes putative contaminating RNAs, confirmed the presence of mt-tRF-Leu^TAA^ inside the mitochondria. Furthermore, the fragment was nearly completely degraded after permeabilization of the mitochondrial membranes with Triton combined with RNAse A, indicating that mt-tRF-Leu^TAA^ was most likely localized in the mitochondria (i.e., in the membranes, intermembrane space or the matrix) (Figure 1I).

### 2.2 The abundance of mt-tRF-Leu^TAA^ in pancreatic islets varies according to the energetic status

**Figure 2:**
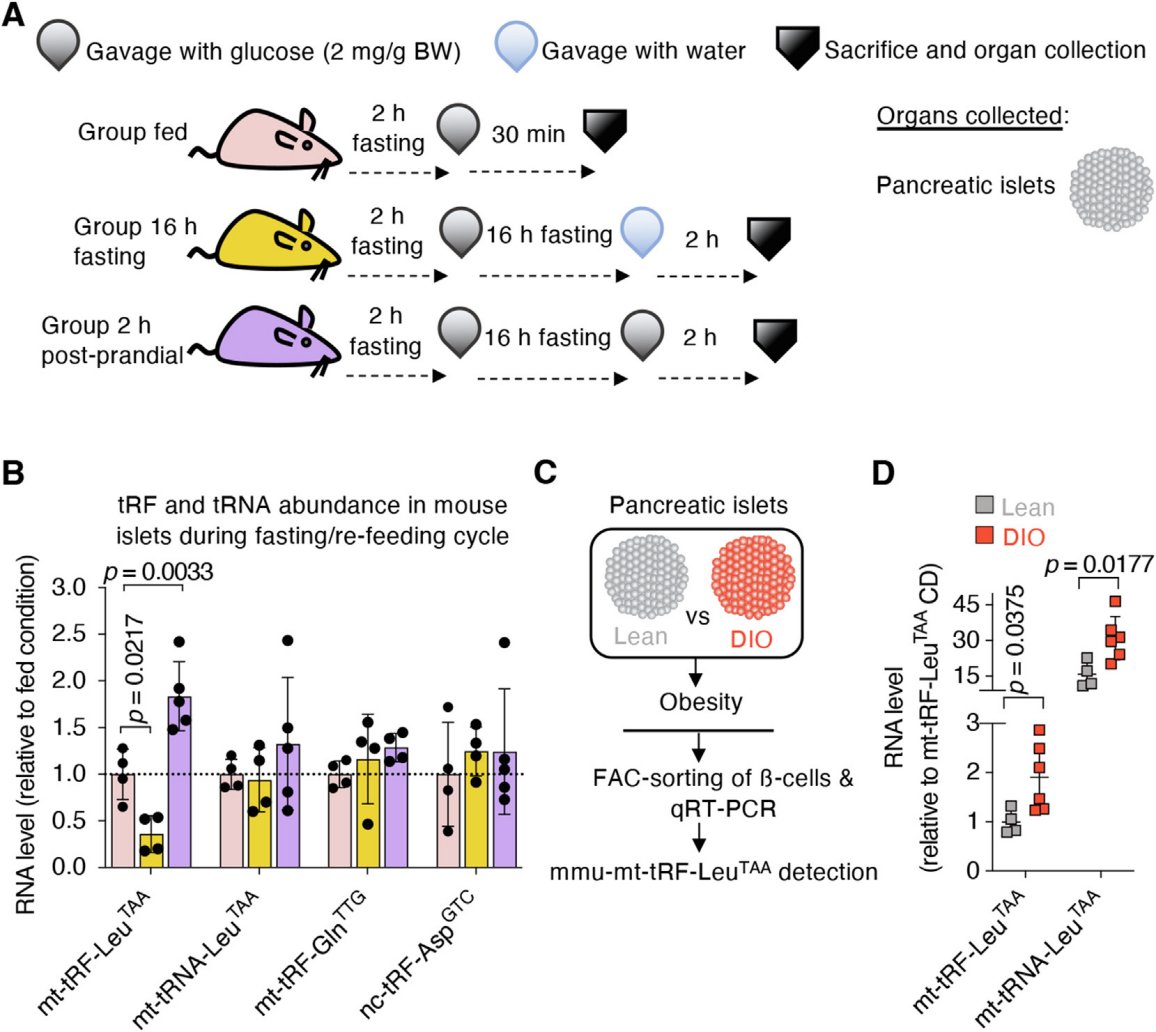
**The abundance of the fragment in the islets varies according to nutritional status.** A. Overview of the experimental procedure with the timing of interventions and nutritional supplies for each group. Pancreatic islets were collected after sacrifice of the mice. B. qRT-PCR measurement of the mitochondrial tRFs mt-tRF-Leu^TAA^ and mt-tRF-Gln^TTG^, the host tRNA mt-tRNA-Leu^TAA^, and a nuclear-encoded tRF nc-tRF-Asp^GTC^ in pancreatic islets during fed, fasting and post-refeeding conditions. Results represent fold changes relative to control with dots representing individual values ± SD, *N* = 4–5, one-way ANOVA followed by Dunnett post-hoc test, *p* ≤ 0.05. C. Schematics of the experimental procedure to assess mt-tRF-Leu^TAA^ levels in FAC-sorted β-cells of diet-induced obese (DIO) mice fed a high-fat diet for 16 weeks vs lean mice fed a control chow diet. D. Measurement of mt-tRF-Leu^TAA^ by real-time PCR. Data are fold changes over control ±SD, *N* = 4–6 with each *N* that consists in the pool of β-cells from 3 lean mice and from 2 DIO mice, unpaired Student's *t*-tests, *p* ≤ 0.05.

The generation of tRFs was reported to be influenced by nutritional status [17]. Here, we observed changes in the level of mt-tRF-Leu^TAA^ in islets of diabetic *db/db* mice and of rats exposed to neonatal protein restriction. These findings raise questions about the regulation of fragment production in response to physiological conditions such as fasting/re-feeding cycles. To investigate this, C57BL/6 mice were subjected to a 2-hour period without food, followed by a single oral dose of 2 mg/g body weight glucose solution to reset the digestive functions and harmonize glycogen stores (referred to as the “fed” group) (Figure 2A). From this group of mice, some underwent a 16-hour fasting followed by water intake (called “16 h fasting”), or glucose intake (called “2 h postprandial”). Blood glucose levels were monitored to ensure effective administration of the carbohydrate (Fig. S2A). Pancreatic islets were collected and analyzed by qRT-PCR (Figure 2A). After 16-hour fasting, mt-tRF-Leu^TAA^ levels in islets decreased by 50 %, while other fragments encoded by the mitochondrial (mt-tRF-Gln^TTG^) and nuclear (nc-tRF-Asp^GTC^) genome and the host mt-tRNA-Leu^TAA^ were unaffected (Figure 2B). Two hours after carbohydrate intake, mt-tRF-Leu^TAA^ levels increased beyond baseline levels in the fed state (Figure 2B). Refeeding did not affect the level of the other fragments or of the host tRNA (Figure 2B). These findings suggest that in β-cells the level of mt-tRF-Leu^TAA^ decreases during fasting when insulin secretion is minimized and raises sharply after carbohydrate intake when insulin release increases. To further explore the link between mt-tRF-Leu^TAA^ and nutritional status, we measured the level of the fragment in β-cells of diet-induced obese (DIO) mice fed a hyperenergetic and hyperlipidic (HFD) diet for 16 weeks (Figures 2C and S2B). This diet leads to the development of obesity, peripheral IR, compensatory insulin hypersecretion, and glucose intolerance [24]. qRT-PCR measurements revealed an increase in mt-tRF-Leu^TAA^ and its host tRNA in FAC-sorted β-cells from DIO mice compared to β-cells from lean mice (Figure 2D). Collectively, these observations suggest that the generation of mt-tRF-Leu^TAA^ in β-cells *in vivo* is regulated by the energetic resources available and coincides with insulin requirements.

### 2.3 The nutrient sensor mTORC1 controls the level of mt-tRF-Leu^TAA^

**Figure 3:**
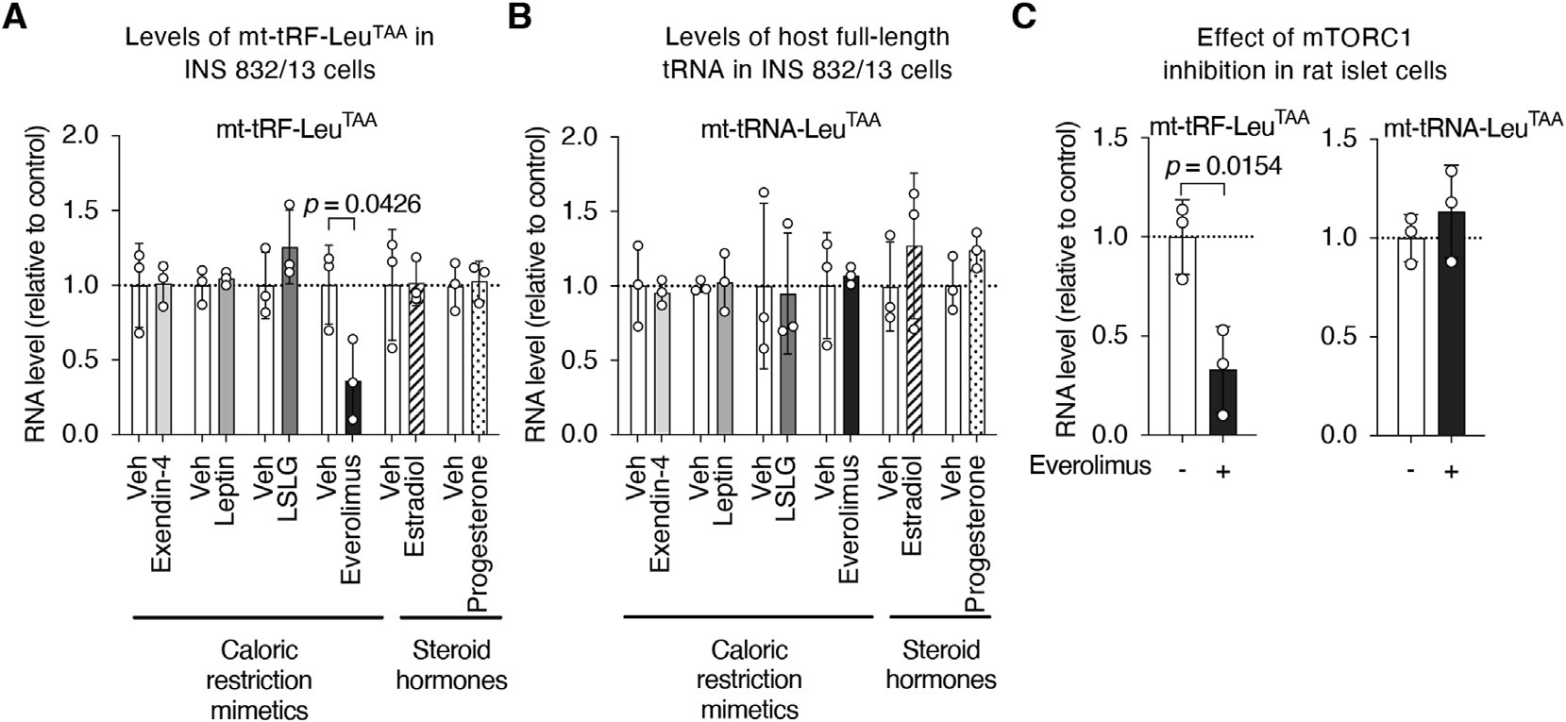
**The inhibition of the nutrient sensor mTORC1 reduces the level of mt-tRF-Leu^TAA^.** A, B. qRT-PCR measurement of mt-tRF-Leu^TAA^ (A) and its host full-length tRNA (B), in insulin-secreting INS 832/13 cells after exposure for 24 h to caloric restriction mimetics (100 nM exendin-4, 50 nM leptin, low serum low glucose (LSLG: 0.01% FBS and 2.8 mM glucose), 40 nM everolimus) or steroid hormones (100 nM estradiol, 100 nM progesterone). Data are expressed as fold changes relative to control condition (vehicle) for each treatment respectively ± SD, *N* = 3, one-sample Student's *t*-tests, *p* ≤ 0.05. C. Measurement by real-time PCR of the levels of mt-tRF-Leu^TAA^ and of its host full-length tRNA (mt-tRNA-Leu^TAA^) in rat islets assessed by real-time PCR in the absence or presence of the mTORC1 inhibitor everolimus (40 nM) for 48 h. Results are expressed as fold changes with dots showing individual values ± SD, *N* = 3, one-sample Student's *t*-tests, *p* ≤ 0.05.

Next, to decipher which molecular pathways might regulate the level of mt-tRF-Leu^TAA^, we exposed INS 832/13 cells to various caloric restriction mimetics [25,26] as well as to steroid hormones. This approach mimics the hormonal changes and the activation of the pathways operating under fasting/re-feeding cycle or during conditions of over- or under-nutrition [27]. We found that the level of mt-tRF-Leu^TAA^ was strongly reduced upon inhibition of mechanistic target of rapamycin complex 1 (mTORC1) with Everolimus (Figure 3A) while the level of its host tRNA (mt-tRNA-Leu^TAA^) was unaffected (Figure 3B). These findings were confirmed in rat islets, where inhibition of mTORC1 with Everolimus treatment for 48 h reduced mt-tRF-Leu^TAA^ levels by more than 70%, while the expression of the host tRNA (mt-tRNA-Leu^TAA^) remained unchanged (Figure 3C). These findings strengthen the notion that the generation of mt-tRF-Leu^TAA^ in β-cells *in vivo* is regulated by energy availability and further highlight its potential to regulate metabolism in line with changes to metabolic status.

### 2.4 Silencing mt-tRF-Leu^TAA^ in pancreatic islets affects mitochondrial metabolism

**Figure 4:**
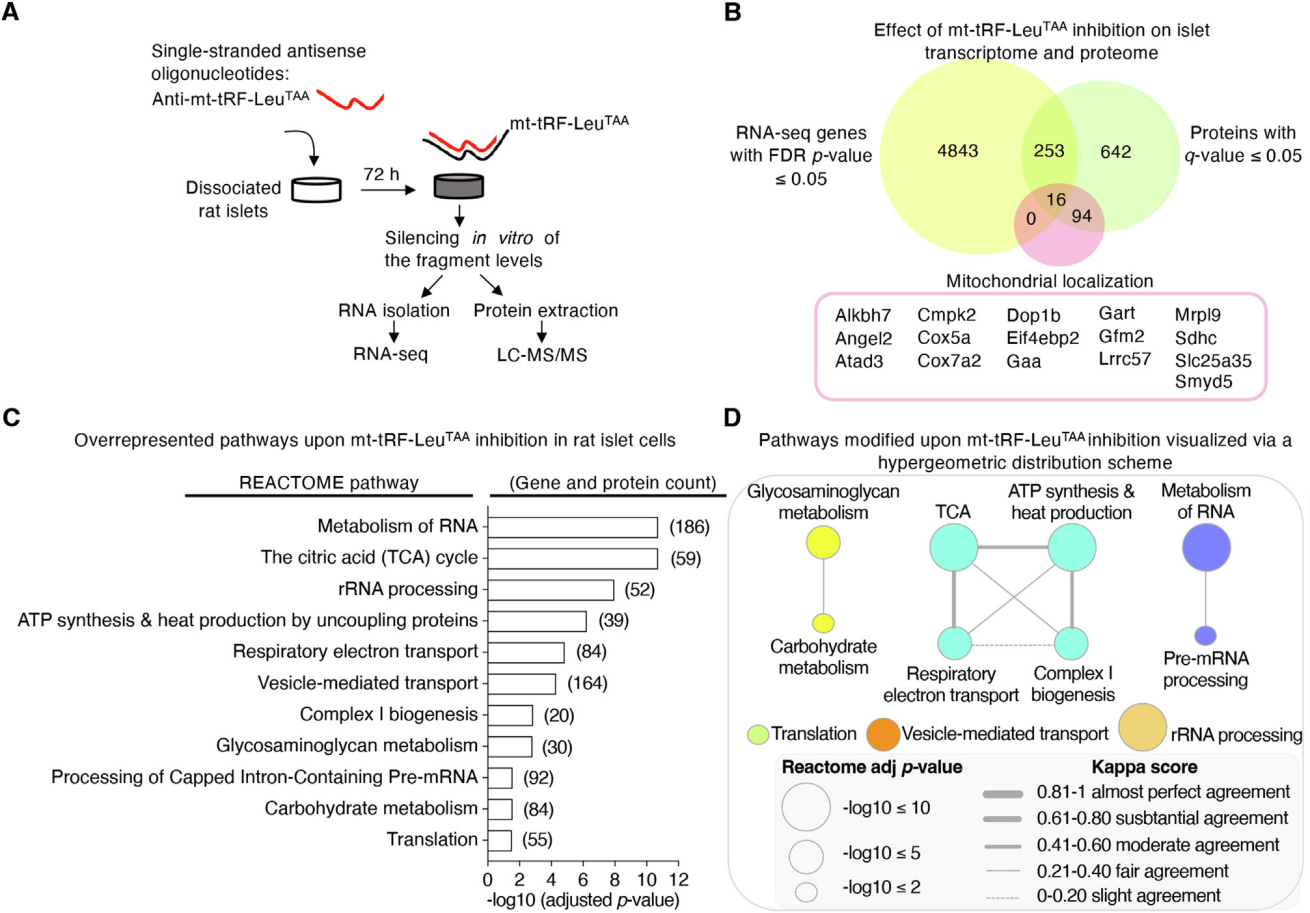
**Silencing mt-tRF-Leu^TAA^ in pancreatic islets negatively impacts on mitochondrial metabolism.** A. To uncover the mechanisms of action of mt-tRF-Leu^TAA^, RNA-sequencing (RNA-seq) and mass spectrometry (LC-MS/MS) were performed on dissociated rat islet cells after silencing the fragment for 72 h B. Venn diagram showing the overlap between significant changes at transcript and protein levels upon mt-tRF-Leu^TAA^ inhibition. Hits that share mitochondrial localization within the complete pool of transcripts (0/4843) and proteins (94/642), as well as amongst the common hits (16/269) are highlighted in pink. *N* = 6, islets from 6 rats for the RNA-seq and *N* = 5, islets from 5 rats for the LC-MS/MS. Islets from the same rat were subdivided into two groups, one for RNA extraction and RNA-seq and the other for protein extraction and LC-MS/MS. False discovery rate (FDR) adjusted *p*-value ≤0.05. C. Pathway enrichment obtained by simultaneous analysis of transcriptome and proteome data sets using REACTOME terms, in the context of mt-tRF-Leu^TAA^ inhibition. The count of genes and proteins in each pathway includes all mRNAs meeting a fold change cutoff of ≥2 and an adjusted *p*-value ≤0.05 (937), and all proteins displaying an FDR adjusted *p*-value cutoff ≤0.05 (642). The analysis encompassed both elevated and reduced hits. D. Enriched REACTOME pathway analysis of mRNA and protein changes upon repression of mt-tRF-Leu^TAA^ using the right-sided hypergeometric test with Benjamini-Hochberg post-hoc *p*-value correction. Terms with a -log10 adjusted *p*-value of 2 or less were visualized in a network layout, where node size corresponds to term -log10 adjusted *p*-value. The proportion of shared genes and proteins between terms was evaluated using the kappa statistic and nodes, with edge width proportional to kappa score.

We next investigated the downstream pathways controlled by mt-tRF-Leu^TAA^. For this purpose, we conducted transcriptomic (GEO accession GSE240395) and proteomic (ProteomeXchange identifier PXD046117) analyses of rat islets in which mt-tRF-Leu^TAA^ was silenced (Figures 4A and S3A; see Figure 8B for the antisense specificity). Inhibition of mt-tRF-Leu^TAA^ led to differential expression of 4843 mRNA transcripts and 642 proteins (cutoff adjusted *p*-value ≤0.05) (Figures 4B and S3B). Among these changes, 269 genes exhibited modifications at both mRNA and protein level. Of note, 16 of these genes are encoded by the nuclear genome but produce proteins that are imported into the mitochondria (Human Protein Atlas: proteinatlas.org). To investigate the impact of mt-tRF-Leu^TAA^ inhibition, we conducted a Gene Ontology (GO) analysis on RNA-sequencing and mass spectrometry data separately (Figs. S3C and S3D). At the transcriptomic level, blockade of the fragment resulted in preferential changes in genes involved in ion transport and in different enzymatic activities, including mitochondrial oxidoreductase (Fig. S3C). At the protein level, an enrichment in genes involved in mitochondrial activities, such as oxidoreductase, ATPase, NADH dehydrogenase and cytochrome-c oxidase, and in hydrogen and oxygen transport was also observed (Fig. S3D). The analysis of data integrating transcriptomic and proteomic changes (combining both up- and down-regulated changes) using REACTOME terms (Figure 4C,D), confirmed an overrepresentation of genes involved in major metabolic pathways, including the TCA cycle and carbohydrate metabolism, with a prominent emphasis on mitochondrial functions such as ATP synthesis, respiratory electron transport, and complex I biogenesis (Figure 4C,D). This analysis also highlighted changes in genes contributing to RNA metabolism, rRNA processing, and vesicle-mediated transport.

**Figure 5:**
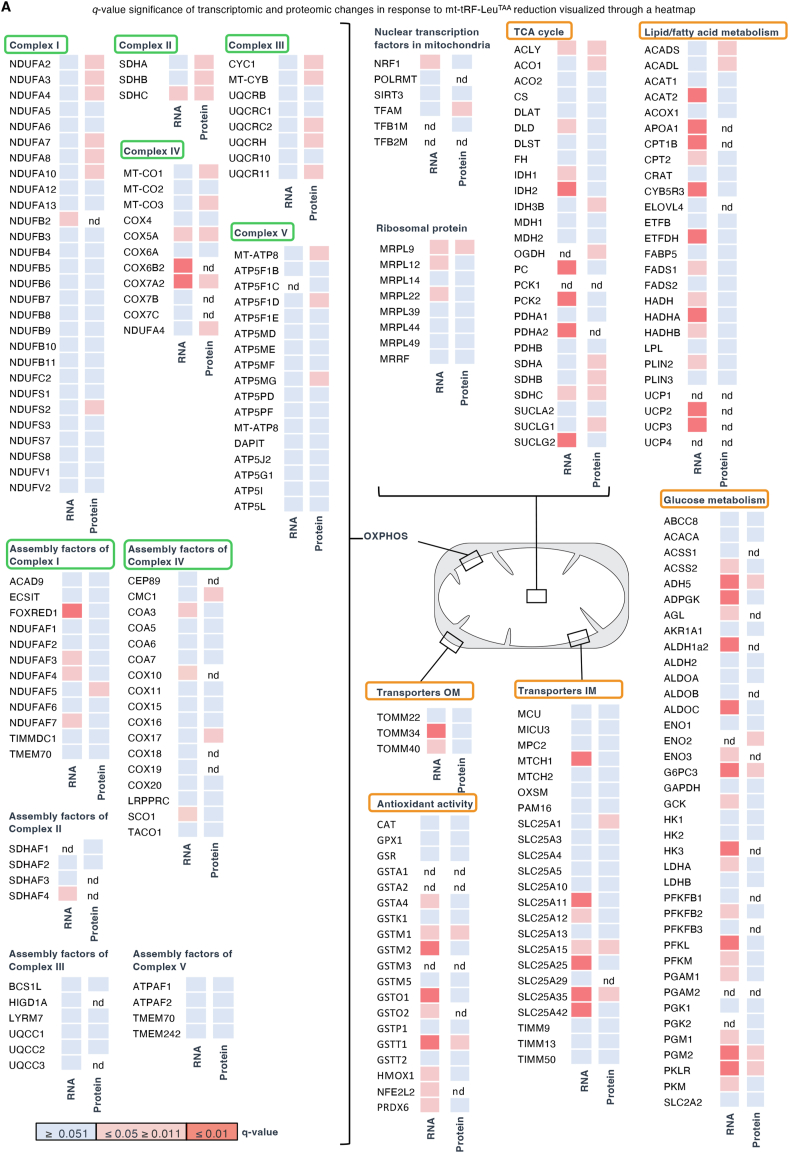
**Bioinformatic analysis of the proteomic and transcriptomic impact of mt-tRF-Leu^TAA^ silencing** A. Heat maps showing the *q*-values of the changes at mRNA and protein levels of the indicated genes observed in islets silenced upon silencing of mt-tRF-Leu^TAA^. Pathways altered predominantly at the protein level are highlighted with a green frame, while pathways impacted mostly at the transcript level are highlighted with an orange frame. Each box indicates the mean expression of *N* = 6 and *N* = 5 samples for mRNAs and proteins, respectively. Colors indicate adjusted *p*-value (*q*-value) computed by the Benjamini-Hochberg method, controlling for false discovery rate (FDR). Non detected genes are indicated (nd).

The heat map of the changes in mRNA and protein levels occurring upon mt-tRF-Leu^TAA^ silencing, revealed relatively few transcriptomic alterations in mitochondrial genes but numerous expression changes in metabolic genes encoded by the nuclear genome. In contrast, at the protein level we observed many differences in key components of the mitochondrial ETS and of their assembly factors (Figure 5A, pathways altered predominantly at the protein level are highlighted with a green frame) but only a handful of differences in metabolic proteins encoded by the nuclear genome. These observations could indicate retrograde signaling from the mitochondria to the nucleus, with alterations of major mitochondrial proteins that subsequently drive a reprogramming of the expression of genes encoded by the nucleus such as TCA cycle, lipid and glucose metabolism, antioxidant signaling, outer membrane (OM) and inner membrane (IM) transporters (Figure 5A, pathways impacted mostly at the transcript level are highlighted with an orange frame).

### 2.5 mt-tRF-Leu^TAA^ interacts with components of the electron transfer system and with other regulators of mitochondrial bioenergetics

**Figure 6:**
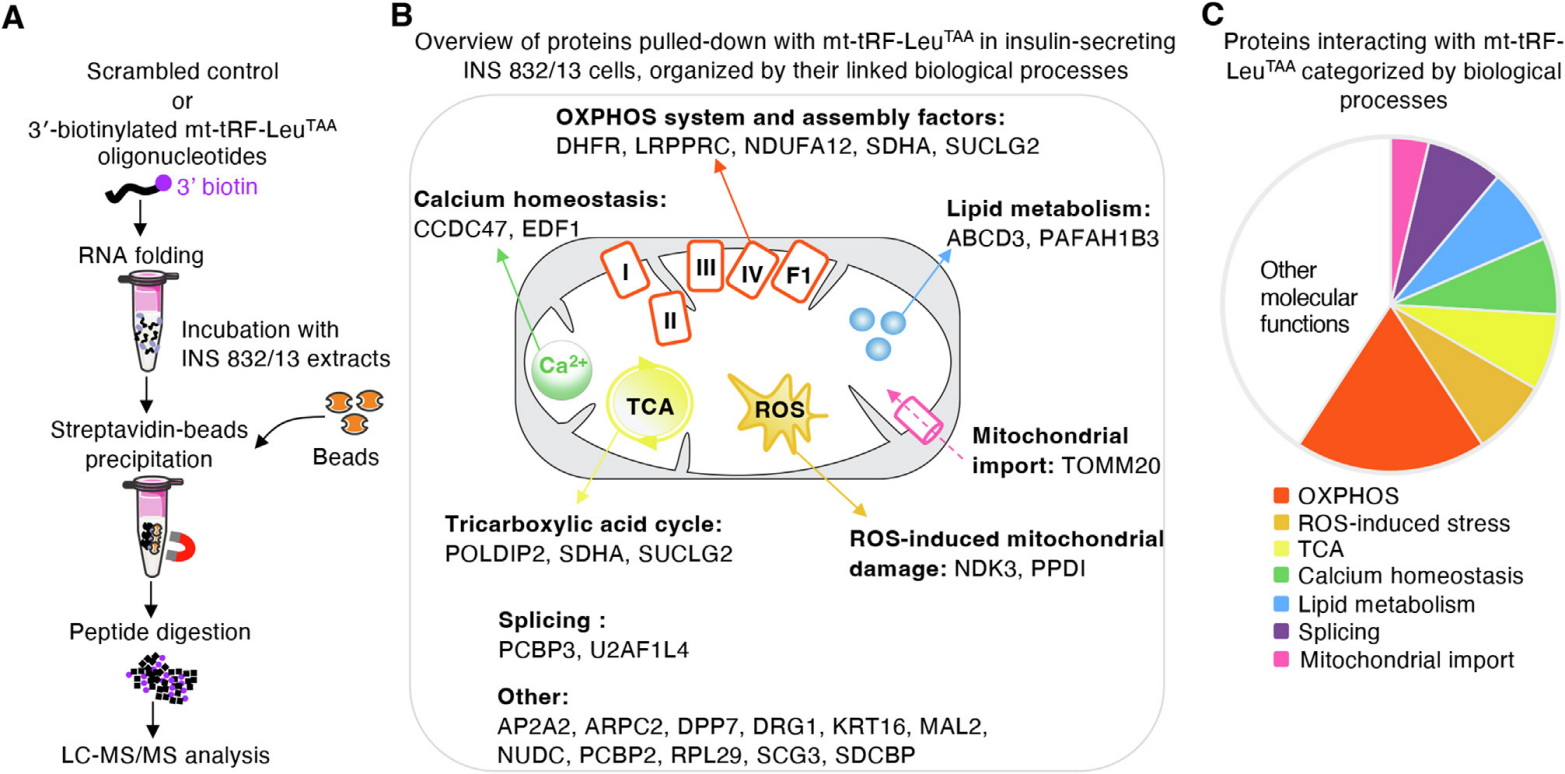
**mt-tRF-Leu^TAA^ interacts with components of the electron transfer system and other regulators of mitochondrial bioenergetics.** A. Representation of the strategy used to identify tRF-interacting proteins by pull-down of 3′biotinylated mt-tRF-Leu^TAA^ mimics or control oligos followed by proteomics. Oligo-specific protein complexes are immunoprecipitated using streptavidin beads and used for quantitative proteomic analysis. B. Scheme showing the proteins bound to mt-tRF-Leu^TAA^ organized by biological processes. Fold-change cutoff ≥6, *t*-test followed by Benjamini-Hochberg method (FDR ≤0.05). C. Alternative representation of Figure 5B. Pie chart illustration of functional classes of proteins captured by mt-tRF-Leu^TAA^ pull-down. D. Scatter plot comparing protein versus transcript abundances of the 24 binding partners of the mitochondrial fragment mt-tRF-Leu^TAA^, utilizing transcriptomic and proteomic data from rat islets with inhibited mt-tRF-Leu^TAA^ (see RNA-seq and LC-MS/MS data presented in Figures 4 and S3). All fold-changes are calculated relative to the control condition. Data points falling on or near the axes intersection have similar expression patterns at both transcript and protein levels. Proteins that display a mitochondrial localization are highlighted. Two-tailed Pearson test.

tRFs have the capacity to bind proteins. Therefore, to identify the mechanisms of action of mt-tRF-Leu^TAA^, we performed pull-down experiments in insulin-secreting INS 832/13 cells followed by mass spectrometry using 3′-biotinylated oligos corresponding to the sequence of mt-tRF-Leu^TAA^ (ProteomeXchange identifier PXD046117) (Figure 6A). The analysis revealed interactions between mt-tRF-Leu^TAA^ and 24 proteins (cutoff fold change ≥6, adjusted *p*-value ≤0.05). Among them are key components of the mitochondrial ETS and major players in OXPHOS activity, including Cyclophilin D, SUCLG2, NDK3, SDHA, LRPPRC and NDUFA12 (Table S1). Binding partners of mt-tRF-Leu^TAA^ are linked to biological processes such as OXPHOS, ROS-induced stress, the TCA cycle, calcium homeostasis, lipid metabolism, RNA splicing and mitochondrial import, which all contribute to the maintenance of mitochondrial metabolism (Figure 6B,C).

### 2.6 mt-tRF-Leu^TAA^ inhibition reduces OXPHOS capacity and limits the mitochondrial membrane potential

**Figure 7:**
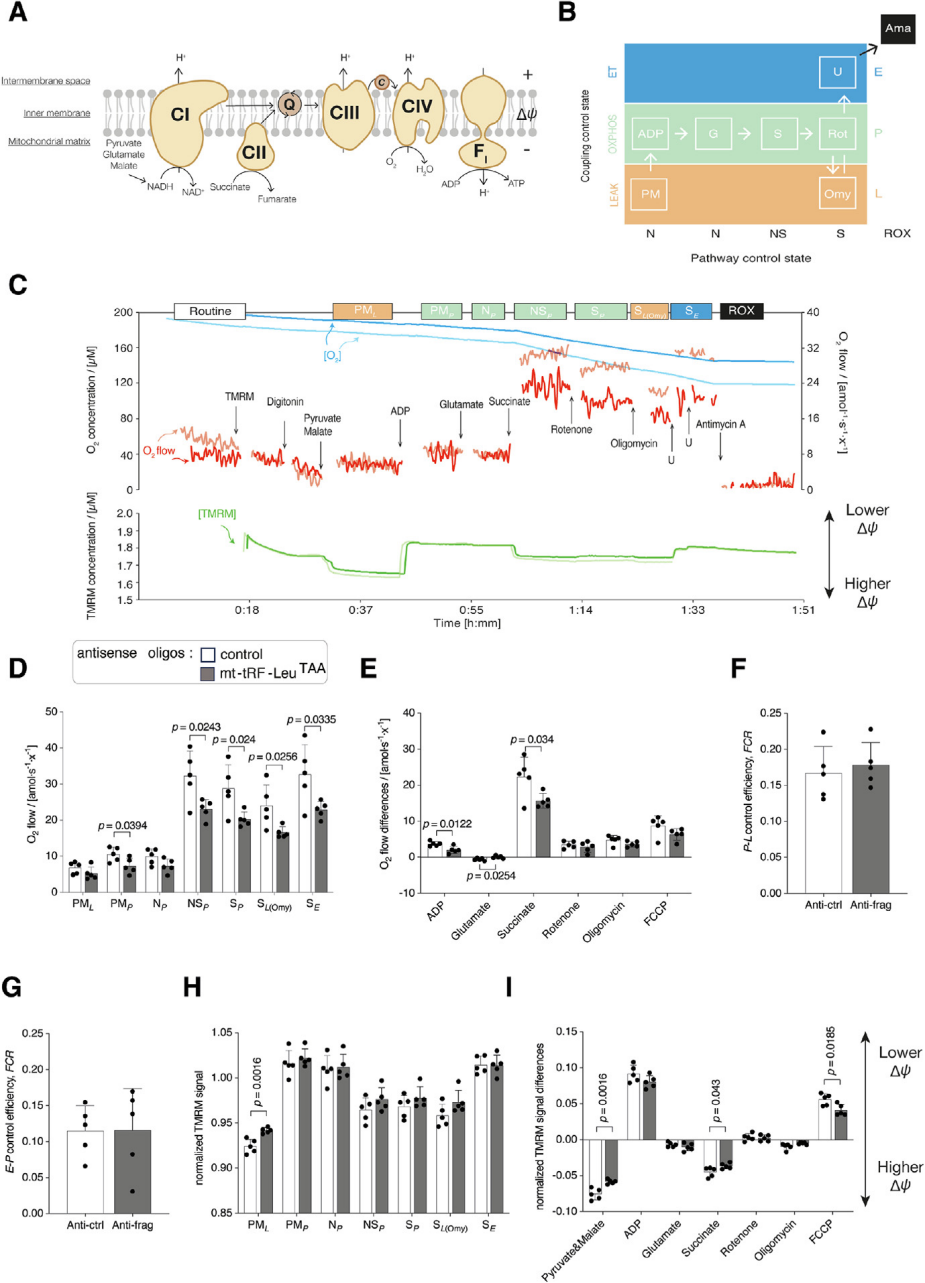
**mt-tRF-Leu^TAA^ inhibition reduces OXPHOS capacity and lowers the mitochondrial membrane potential.** A. Schematic representation of mitochondrial electron transfer from NADH-linked substrates through Complexes CI, CIII, and CIV (N-pathway) and from succinate through CII, CIII, and CIV (S-pathway). Pyruvate, glutamate and malate (PGM) support the N-pathway through CI into the Q-cycle (Q); succinate provides electrons via CII into the Q junction. Electrons are transferred from CIII via cytochrome *c* (*c*) to CIV where O_2_ is reduced to H_2_O. H^+^ ions are pumped across the mt-inner membrane by CI, CIII, and CIV to generate an electrochemical potential difference across the mt-inner membrane, which drives phosphorylation of ADP to ATP by F_O_F_1_-ATPase. B. Coupling/pathway control diagram showing the sequential steps in the substrate-uncoupler-inhibitor titration protocol with different coupling states. Pyruvate P; Glutamate G; Malate M; Adenosine diphosphate ADP; Succinate S; Uncoupler U; Rotenone Rot; Oligomycin Omy; Antimycin A Ama; LEAK respiration *L*; Oxidative phosphorylation OXPHOS capacity *P*; Electron transfer capacity *E*; Residual oxygen consumption ROX. C. Original traces of oxygen concentration (blue traces), oxygen flow (red traces) and TMRM concentration (green traces) from experiments with permeabilized rat pancreatic islet cells. Islets have been dispersed into single cells and transfected for 72 h with antisense oligonucleotides to reduce mt-tRF-Leu^TAA^ levels (darker traces) versus control oligos (lighter traces). D. Respiratory capacities in permeabilized rat pancreatic islet cells normalized for protein mass. *N* = 5 per group. E. Specific effects of substrates, uncouplers and inhibitors on O_2_ ﬂuxes in permeabilized rat pancreatic islet cells normalized for protein mass. *N* = 5 per group. F. Succinate-pathway OXPHOS coupling efficiency in permeabilized rat pancreatic islet cells. *N* = 5 per group. G. Succinate-pathway electron transfer excess capacity in permeabilized rat pancreatic islet cells. *N* = 5 per group. H. Normalized TMRM signal in permeabilized rat pancreatic islet cells normalized for cell count. *N* = 5 per group. I. The specific effects of substrates, uncouplers and inhibitors on the normalized TMRM signal in permeabilized rat pancreatic islet cells normalized for cell count. *N* = 5 per group. The bars represent the means, the error bars the standard deviations, and the dots are individual values. Unpaired Student's *t*-tests were performed, *p* ≤ 0.05.

Given the key ETS subunits and assembly factors identified as binding partners of mt-tRF-Leu^TAA^, we investigated the impact of mt-tRF-Leu^TAA^ inhibition on the function of the mitochondrial electron transfer system (Figure 7A). To do so, we used permeabilized rat pancreatic islet cells and an established substrate-uncoupler-inhibitor titration protocol for high-resolution respirometry [28] that allows simultaneous measurement of mitochondrial respiration with mitochondrial membrane potential (Δ*Ψ*_mt_) in different pathway- and coupling-control states (Figure 7B,C). The NADH- and Succinate-linked pathways, which are two major electron transfer pathways were studied both separately and in combination (Figure 7A).

Our results revealed that silencing mt-tRF-Leu^TAA^ reduced mitochondrial OXPHOS capacity in rat islet cells by ∼30% (Figures 7D, S4D). This deficit was observed with linear electron flow into the Q-junction from the NADH-linked pathway (N_*P*_) and the Succinate-linked pathway (S_*P*_), and convergent electron flow from both pathways (N_*SP*_; Figures 7D,E, S4D,E).

To gain further insights from our OXPHOS analysis we calculated flux control ratios to obtain detailed fingerprints of mitochondrial respiratory control [29]. Our analyses showed high proton leak in rat pancreatic islets (evidenced by low OXPHOS coupling efficiencies; Figures 7F, S4F) - the hallmark of islet cell mitochondrial bioenergetics [30,31]. However, in response to mt-tRF-Leu^TAA^ silencing there were no changes in coupling control compared with control oligos (Figures 7F,G, S4F). Given the decrease in OXPHOS capacity (Figure 7D) occurred without changes in *P*-*L* control efficiency (correcting OXPHOS capacity for LEAK respiration determines the oxygen consumption strictly linked to ADP phosphorylation, i.e., ATP generation; Figures 7F, S4F) this means that mt-tRF-Leu^TAA^ silencing reduces the capacity of islet mitochondria to phosphorylate ADP to ATP. Our analyses also showed that this was not due to direct effects on the phosphorylation system (i.e ATP synthase; Figure 7G) or proton leakage (Figures 7F, S4F). Thus, mt-tRF-Leu^TAA^ likely influences mitochondrial ATP generating capacity by effecting specific electron transfer pathways to oxygen. This notion is further supported by our simultaneous measurements of Δ*Ψ*_mt_ with respiration, which revealed a concurrent reduction in Δ*Ψ*_mt_ with reduced respiratory capacities in response to Succinate (i.e., through Complex II (CII), Complex III (CIII), and Complex IV (CIV); Figures 7H, S4G). This observation suggests that the reduction in oxidative phosphorylation capacity was predominately driven by the Succinate (via CII) pathway which is the main contributor to OXPHOS capacity in rat islets (Figures 7D,E).

Taken together, the reduction in Δ*Ψ*_mt_ and OXPHOS capacity without changes in coupling control, demonstrate that mt-tRF-Leu^TAA^ silencing reduces islet cells capacity to chemiosmotically couple the oxidation of reduced fuel substrates (e.g., from glucose by electron transfer to oxygen), to the phosphorylation of ADP to ATP.

### 2.7 Inhibition of mt-tRF-Leu^TAA^ impairs glucose-induced insulin secretion independently from K_ATP_ channel closure

**Figure 8:**
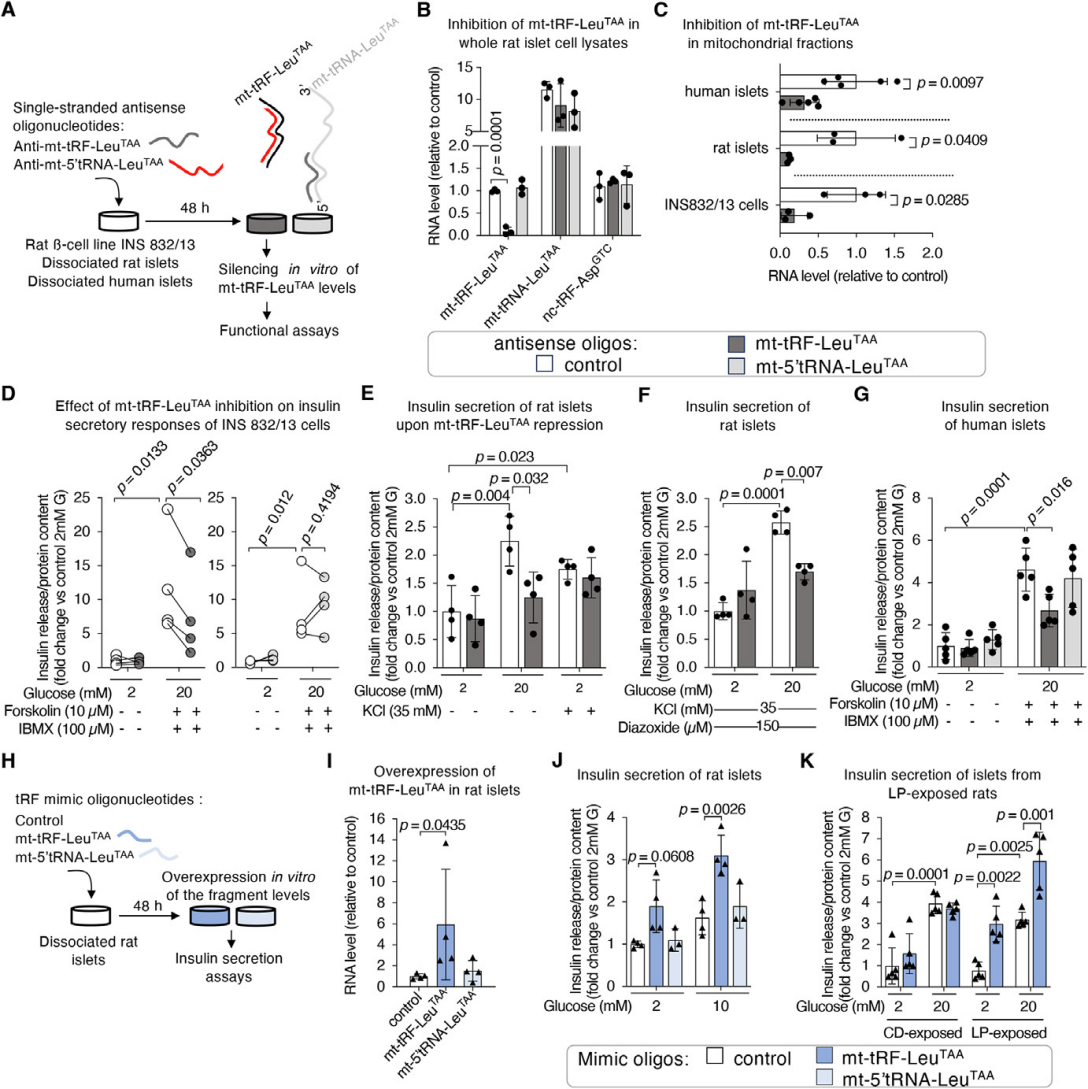
**Inhibition of mt-tRF-Leu^TAA^ impairs glucose-induced insulin secretion by a pathway independent of K_ATP_ channel closure while its overexpression enhances insulin secretion.** A. Schematic representation of the experimental strategy to modulate the level of the fragment mt-tRF-Leu^TAA^*in vitro* in order to mimic its reduction observed *in vivo* under pre-diabetic conditions. Synthetic single-stranded antisense oligonucleotides correspond to the complementary sequence of the fragment of interest (anti-mt-tRF-Leu^TAA^, 38 nucleotide-long). Antisense oligonucleotides targeting the 5′ region of the host mt-tRNA-Leu^TAA^ (anti-mt-5′tRNA-Leu^TAA^, 29 nucleotide-long) are used as a control to check the specificity of our inhibitory approach. Rat insulinoma cell line INS 832/13, adult rat and human islets were dispersed and transfected with antisense oligonucleotides to reduce mt-tRF-Leu^TAA^ levels. 48 h later, the capacity of β-cells to release insulin was assessed. B. Confirmation by qRT-PCR that anti-mt-tRF-Leu^TAA^ specifically reduces the levels of mt-tRF-Leu^TAA^ without affecting its host full-length tRNA (mt-tRNA-Leu^TAA^) nor a cytosolic fragment (nc-tRF-Asp^GTC^). Data are the mean ± SD expressed as fold changes relative to control, *N* = 3, one-way ANOVA and Dunnett post-hoc test, *p* ≤ 0.05. C. qRT-PCR validation of mt-tRF-Leu^TAA^ depletion in mitochondrial preparations from rat and human islets, and from INS 832/13 cells. Data are expressed as fold changes ±SD, *N* = 3–5, unpaired Student's *t*-tests, *p* ≤ 0.05. D. Insulin release from INS 832/13 cells transfected with a control oligonucleotide or antisense against mt-tRF-Leu^TAA^ (left panel) or against mt-5′tRF-Leu^TAA^ (right panel) after 45 min exposure to 2 mM glucose or to 20 mM glucose, 10 μM forskolin, and 100 μM IBMX as a cocktail for stimulatory condition. Insulin secretion is normalized to protein content for each group (Fig. S5B) and is represented as the fold changes vs. control basal (2 mM glucose) condition. Data are shown as individual values, *N* = 4, two-tailed paired *t*-tests, *p* ≤ 0.05. E. Insulin was measured by ELISA and normalized to protein content. After a 30 min pre-incubation at 2 mM glucose, the cells were stimulated for 45 min with 2 mM or 20 mM glucose in the presence or absence of 30 mM KCl. High (20 mM) glucose markedly augments insulin secretion through glucose metabolism by oxidative glycolysis and the rise of derived-metabolic signals. Depolarization of β-cells with KCl raises intracellular calcium concentration and triggers insulin secretion at low (2 mM) glucose. Insulin content was unchanged (Fig. S5C). Results are expressed as fold changes of 4 independent experiments with error bars indicating SD, *N* = 4, two-way ANOVA followed by a Tukey post-hoc test, *p* ≤ 0.05. F. mt-tRF-Leu^TAA^ inhibition affects insulin secretion independently of K_ATP_ channel closure. After a 30 min pre-incubation at 2 mM glucose, cells were stimulated for 45 min with 2 mM or 20 mM glucose in the presence or absence of 30 mM KCl and 150 μM diazoxide. The addition of diazoxide (a K_ATP_ channel activator) to KCl allows the study of GSIS under conditions of high cytosolic Ca^2+^ entry through a K_ATP_ channel-independent pathway. Insulin was measured by ELISA and normalized to protein content. Insulin content was unchanged (Fig. S5C). Results are expressed as fold changes ±SD, *N* = 4, two-way ANOVA and Tukey post-hoc test, *p* ≤ 0.05. G. Insulin release of dissociated human islets upon inhibition of mt-tRF-Leu^TAA^ or of the 5′ region of the host tRNA in response to 2 mM glucose or to 20 mM glucose, 10 μM forskolin, and 100 μM IBMX for 45 min. Insulin secretion is normalized by the protein content for each group (Fig. S5D) and is represented as the fold change vs. control basal (2 mM glucose) condition with error bars indicating SD, *N* = 5, two-way ANOVA with a Tukey post-hoc test, *p* ≤ 0.05. H. Experimental approach *in vitro* for the overexpression of mt-tRF-Leu^TAA^ using mimic sequences of the fragment or of the 5′ region of the host tRNA as a negative control (mt-5′tRNA-Leu^TAA^) by transfection of dissociated islet cells for 48 h. I. Validation of the overexpression of mt-tRF-Leu^TAA^ by qRT-PCR. Data represent mean of 4 independent experiments ±SD, unpaired Student's *t*-tests, *p* ≤ 0.05. J. Assay *in vitro* of insulin secretion in response to 2 mM or 20 mM glucose represented as the fold change vs. control basal (2 mM glucose) condition. Insulin release was normalized by insulin content which was unchanged (Fig. S5I). Triangles display individual values and error bars indicate SD, *N* = 5 independent experiments. Two-way ANOVA and Šidák correction, *p* ≤ 0.05. K. Assay *in vitro* of insulin release in response to 2 mM or 10 mM glucose represented as the fold change vs. control basal (2 mM glucose) condition. Insulin release was normalized by insulin content which was unchanged (Fig. S5J). Triangles show individual values and error bars indicate SD, *N* = 3–4 independent experiments. Two-way ANOVA followed by a Tukey post-hoc test, *p* ≤ 0.05.

The results showing that mt-tRF-Leu^TAA^ plays a key role in regulating mitochondrial ATP generation in islet cells raises the question if this fragment can regulate insulin secretion. To address this question, we transfected INS 832/13 cells as well as rat and human islet cells with antisense oligonucleotides (Figure 8A). These oligos (anti-tRF-Leu^TAA^) specifically decreased the levels of the fragment by more than 95% without affecting the level of full-length mt-tRNA-Leu^TAA^ nor the levels of a cytosolic fragment (nc-tRF-Asp^GTC^) used as a negative control (Figure 8B). Pull-down investigations using biotinylated anti-tRF-Leu^TAA^, revealed that the antisense oligo binds specifically to the fragment and not to the host tRNA (Fig. S5A), probably explaining the selectivity of the effect. To further confirm the specificity of anti-tRF-Leu^TAA^, we generated antisense oligos targeting the 5′ region of the host tRNA (anti-5′tRNA-Leu^TAA^). Anti-5′tRNA-Leu^TAA^ had no effect on the levels of mt-tRF-Leu^TAA^, the full-length mt-tRNA-Leu^TAA^, or of nc-tRF-Asp^GTC^ (Figure 8B). We verified that the antisense oligos successfully reached the mitochondrial compartment by specifically reducing the levels of mt-tRF-Leu^TAA^ in MF (Figure 8C). Silencing mt-tRF-Leu^TAA^ in INS 832/13 cells exposed to high glucose and to the cAMP-raising agents IBMX and forskolin, resulted in impaired insulin release. In contrast, anti-5′tRNA-Leu^TAA^ had no effect on insulin release in both basal and stimulated states (Figure 8D).

In dispersed rat islet cells, reduction of mt-tRF-Leu^TAA^ levels resulted in defective insulin secretion in response to glucose (GSIS), while insulin release triggered by depolarizing KCl concentrations remained unaffected (Figure 8E). This suggests an impairment in glucose metabolism rather than a defect in K_ATP_ channel-dependent Ca^2+^ signaling or in the exocytotic machinery. To investigate the role of mt-tRF-Leu^TAA^ in regulating the amplifying K_ATP_-independent pathway of GSIS, we exposed rat islet cells to a stimulatory concentration of KCl in the presence of diazoxide. This K_ATP_-channel opener allows the assessment of GSIS at high cytosolic Ca^2+^ independently of K_ATP_ channel closure. This experiment confirmed that mt-tRF-Leu^TAA^ silencing compromised the effectiveness of glucose metabolism and ATP production in stimulating Ca^2+^ rise and thereafter the coupling with insulin granule exocytosis (Figure 8F). The involvement of the fragment in GSIS was also confirmed in human β-cells (Figure 8G). Insulin secretion was normalized to insulin content which is not affected by mt-tRF-Leu^TAA^ silencing (Figures S5B, S5C and S5D).

We also investigated the potential impact of mt-tRF-Leu^TAA^ inhibition on survival and proliferation of insulin-secreting cells. We found no changes in rat and human β-cell survival under basal or cytokine-induced pro-inflammatory conditions (Figs. S5E, S5F, S5G), nor in the proliferation capacity of INS 832/13 cells (Fig. S5H). Collectively, these findings strongly indicate that the mitochondrial fragment mt-tRF-Leu^TAA^ regulates insulin release by controlling the ability of islet cells to efficiently couple glucose metabolism to a rise in ATP, while importantly highlighting the conservation of this regulatory function between the orthologous fragments in rat and human.

### 2.8 Overexpression of mt-tRF-Leu^TAA^ enhances insulin secretion

Enhancing β-cell insulin secretion capacity remains a critical challenge in the reversal of β-cell dysfunction and/or loss in diabetes. We found that elevating the levels of mt-tRF-Leu^TAA^ in rat islet cells (Figure 8H,I), enhanced insulin secretion in response to intermediate glucose concentrations (Figure 8J), while overexpression of the 5′ region of the whole mt-tRNA-Leu^TAA^ had no significant impact (Figure 8J). Furthermore, in conditions associated with impaired mitochondrial metabolism such as in islets of LP-exposed rats, overexpression of mt-tRF-Leu^TAA^ increased insulin secretion in response to low- and high-glucose (Figure 8K). Cellular insulin content remained unaltered in these conditions (Figs. S5I and S5J). These findings emphasize the requirement of the mitochondrial fragment to achieve optimal β-cell secretory functions.

## 3 Discussion

Our study establishes a yet unrevealed connection between the regulation of the mitochondrial tRNA-derived fragment mt-tRF-Leu^TAA^ in pancreatic islets with metabolism-insulin secretion coupling. mt-tRF-Leu^TAA^ shows enrichment within islet mitochondria and exhibits variation based on nutritional status and mTORC1 activation. Through its interaction with electron transfer system complexes, it governs mitochondrial respiration and insulin secretion in both rat and human β-cells.

Lower levels of mt-tRF-Leu^TAA^ in β-cells are linked to reduced insulin secretion capacity in mice upon 16-hour fasting, in obese diabetic *db/db* mice, and in adult rats exposed to fetal and postnatal protein deficiency. All these animal models are characterized by reduced insulin secretion in response to glucose, either as an adaptation to fasting or because of impaired β-cell function [[18], [19], [20]]. Conversely, increased insulin demand and enhanced insulin secretion, as seen after fasting/re-feeding or in mice fed a HFD, is associated with a rise in the levels of mt-tRF-Leu^TAA^ [32,33]. In agreement with these observations, mt-tRF-Leu^TAA^ inhibition in islet cells causes impairments in mitochondrial ATP generation capacities and impaired GSIS while overexpression of the fragment increases β-cell capacity to release insulin.

In our study, we were able to silence mt-tRF-Leu^TAA^ without affecting the level of the parental tRNA. Pull-down of anti-tRF-Leu^TAA^ led to a selective enrichment of mt-tRF-Leu^TAA^ but not of the full-length tRNA, potentially explaining the specificity of anti-tRF-Leu^TAA^. A possible explanation for this finding could rely on differences in the conformation between the fragment and its parental tRNA. In fact, tRNAs assume a highly structured three-dimensional L-shape conformation [34] that may hinder the binding of anti-tRF-Leu^TAA^. In contrast, once cleaved the fragment is likely to unwind and assume an unfolded conformation more prone to interact with other RNAs or protein-binding partners, including antisense molecules. This would emphasize the importance of generating tRNA-derived fragments that interact with specific binding partners and acquire functional properties at least partially independent from their hosting tRNAs [11,12].

Bioinformatic analysis of the proteomic impact of mt-tRF-Leu^TAA^ silencing mapped the changes to proteins involved in TCA cycle, ATP synthesis, ETS, and carbohydrate metabolism. Transcriptomic analysis showed slight variations in mitochondrial genes, yet substantial alterations were found in the expression of metabolic genes encoded by the nuclear genome. Reprogramming of cytosolic metabolic pathways such as lipid and glucose metabolism, antioxidant signaling, OM and IM transporters may result from signaling from the mitochondria to the nucleus [35]. This may explain why 72 h post-transfection mainly a mitochondrial remodeling in proteomics and only cytosolic transcriptomic changes are observed. Mitochondrial non-coding RNAs, like double-stranded RNAs, can trigger mitochondrial retrograde signaling upon their export and re-localization to the cytosol [36]. Our results do not indicate any potential export of mt-tRF-Leu^TAA^ to the cytosol. Instead, the fragment seems predominantly concentrated in mitochondrial fractions. The speculated mitochondrial-cytosolic communication triggered by mt-tRF-Leu^TAA^ could involve its interaction with proteins aiding in mitochondrial signal export to the cytosol, despite the current lack of understanding of this mechanism [37]. The phenotype and molecular reshuffling related to mt-tRF-Leu^TAA^ inhibition likely stem from its direct interactions with conserved ETS proteins and assembly factors, revealed by MS analysis of pulled-down proteins, including Cyclophilin D, SUCLG2, SDHA, LRPPRC, and NDUFA12 [[38], [39], [40]]. The expression of these factors is not majorly affected by mt-tRF-Leu^TAA^ silencing, suggesting that the fragment modulates the function or activity of ETS proteins and assembly factors rather than their expression levels. Binding to SDHA, could place mt-tRF-Leu^TAA^ as potential key regulator for the Succinate-pathway, generating the electric component (Δ*Ψ*_mt_) of the protonmotive force for ATP production [3]. The modulation of the activity of ETS complexes aligns with respirometry results obtained upon mt-tRF-Leu^TAA^ inhibition.

Using respirometry, we and others have noted that in rat islets, the coupling efficiency between OXPHOS and ATP production primarily relies on the Succinate-linked pathway [3,30,31]. Blocking mt-tRF-Leu^TAA^ reduced OXPHOS capacity and compromised the coupling of mitochondrial oxygen consumption and ATP regeneration, primarily affecting oxygen consumption related to NADH and Succinate, the substrates of complex I and II, respectively. Interestingly, the reduction of 30% in insulin secretion in response to 20 mM glucose is proportional to the reduced OXPHOS capacity through the combined NADH- and Succinate-linked pathways (NS_P_). The Δ*Ψ*_mt_ is another important physiologic mitochondrial parameter as it contributes to the generation of protonmotive force necessary for ATP regeneration by OXPHOS and enables mitochondrial Ca^2+^ entrance, crucial for insulin secretion [3,41]. Δ*Ψ*_mt_ limitation, induced by mt-tRF-Leu^TAA^ silencing, likely leads to reduced ATP production and impaired β-cell bioenergetics. However, mt-tRF-Leu^TAA^ inhibition did not affect mRNA and protein levels of lactate dehydrogenase (LDH), suggesting that the decrease in OXPHOS capacity does not induce a compensatory shift toward anaerobic metabolism through LDH [3].

The ongoing debate in diabetes research questions whether IR or insulin hypersecretion is the primary cause of T2D [42]. Initially considered a beneficial compensatory response, chronically elevated GSIS is now seen as potentially harmful, contributing to β-cell exhaustion, promoting gluconeogenesis and hepatic lipogenesis, exacerbating “nutrient-induced metabolic stress” and IR in skeletal muscle, liver, and adipose tissue [42]. Conversely, loss of insulin sensitivity, once viewed negatively, is now seen as protective [42]. Thus, elevated levels of mt-tRF-Leu^TAA^ in the islets of DIO mice might contribute to insulin hypersecretion. Limiting mt-tRF-Leu^TAA^ production may serve as a protective measure against T2D by mitigating excessive GSIS. Additionally, elevated fasting levels of five amino acids, including leucine, have been linked to T2D development in normoglycemic individuals [43]. We observed increased host mt-tRNA-Leu^TAA^ levels in the islets of DIO mice. The rise in host mt-tRNA-Leu^TAA^ levels might lead to a concurrent increase in mt-tRF-Leu^TAA^ production, as evidenced in the FAC-sorted β-cells of DIO mice prone to diabetes. However, the confirmation of increased plasma levels of leucine in these mice remains to be investigated.

Pharmacological inhibition of mTORC1 *in vitro* reduces mt-tRF-Leu^TAA^ levels, leading to decreased insulin secretion and aligning with mTORC1 inhibitors effects [44]. Even though, the endoribonuclease(s) responsible for mt-tRNA-Leu^TAA^ cleavage are yet to be identified, we can speculate that mTORC1 may contribute to modulating the level of mt-tRF-Leu^TAA^ in response to changes in the nutritional state. Drugs like metformin or low-dose rapamycin seemingly enhance metabolic profiles in diabetic patients by partly restraining mTORC1 signaling [45]. Further exploration of the link between mTORC1 activation and mt-tRF-Leu^TAA^ levels may hold promise for the discovery of novel therapies.

mt-tRF-Leu^TAA^ is generated from the mt-tRNA-Leu^TAA^ gene (sometimes also referred to as mt-tRNA-Leu^UUR^) [46]. A point mutation leading to an A-to-G transition (A3243G) in this gene leads to a defective taurine-containing modification of a uridine in the anticodon wobble position. The lack of this modification is linked to both Mitochondrial Encephalomyopathy, Lactic Acidosis, and Stroke-like Episodes (MELAS) and Maternally Inherited Diabetes and Deafness (MIDD). The A3243G mutation markedly decreased the expression of mt-tRNA-Leu^TAA^-derived mt-tRFs in fibroblasts from MELAS patients [47]. Moreover, an osteosarcoma cell line with patient-derived A3243G mutated mt-DNA was found to display decreased glucose oxidation and diminished Δ*Ψ*_mt_, ultimately resulting in reduced ATP generation [48]. Our findings, showing impairments in mitochondrial oxidative phosphorylation after mt-tRF-Leu^TAA^ silencing in insulin-secreting cells, suggest that further investigations are warranted to determine whether a potential link exists between the depletion of mt-tRNA-Leu^TAA^-derived fragments observed in patients with the A3243G mutation, β-cell dysfunction, and T2D development.

Our study demonstrates that the mitochondrial tRNA-derived fragment mt-tRF-Leu^TAA^, varies in pancreatic islets and β-cells in response to changes in the nutritional status. Through interactions with different mitochondrial regulators, the fragment affects mitochondrial respiration and modulates rodent and human insulin secretion capacities of pancreatic β-cells. Since the sequence and function of mt-tRF-Leu^TAA^ is overall preserved in humans, our findings underscore the importance of investigating the regulation of this tRNA-derived fragment in individuals with obesity and diabetes.

## 4 Limitations of the study

Some limitations of the study are worth noting. In view of our findings, it would have been very interesting to assess whether adjusting mt-tRF-Leu^TAA^ levels in diabetes-prone animal models could prevent diabetes onset. However, mt-tRF-Leu^TAA^ is generated from an essential mitochondrial tRNA and it is currently not possible to design experiments to modulate selectively the fragment *in vivo* in specific metabolic tissues without affecting the level of the full-length tRNA. Moreover, this study focused on one specific tRNA-derived fragment and does not exclude that other nuclear- or mitochondrially-encoded tRFs may also contribute to the onset of diabetes. However, selective silencing of mt-tRFs displaying changes in diabetes-susceptible rodents without affecting the full-length tRNA may be challenging, in particular for long fragments.

We have demonstrated that mt-tRF-Leu^TAA^ interacts with several components of the ETS and can modulate the function of these mitochondrial complexes. The elucidation of the precise molecular mechanisms causing these effects would require the reconstitution of the mitochondrial complexes and a detailed evaluation of the impact of the fragment on the activity of each ETS component. Despite nucleotide sequences partially overlapping with full-length mt-tRNA-Leu^TAA^, mt-tRF-Leu^TAA^ appears to interact with specific protein-binding partners. This may be linked to differences in the three-dimensional conformation between the highly structured full-length tRNA and the tRNA fragment. This assumption would need to be confirmed by structural data about the conformation of the fragment.

In this study, we compared relative fluctuations of mt-tRF-Leu^TAA^ levels across various physiopathological settings. We cannot exclude changes in absolute mt-tRF-Leu^TAA^ levels following isolation and culture of pancreatic islets. *In situ* hybridization techniques would potentially permit to analyze the level of mt-tRF-Leu^TAA^ on pancreatic slices avoiding the isolation and culture procedures and to confirm the enrichment of the fragment in mitochondria. However, at present the detection and quantification of tRFs by *in situ* hybridization is not yet feasible. In fact, a major obstacle of this method is given by the necessity to denature the target and disrupt its tertiary structure. This would allow the oligonucleotide designed to detect mt-tRF-Leu^TAA^ to bind also parental mt-tRNA-Leu^TAA^ which is 10–20 times more abundant than the fragment, compromising the specificity of the probe.

## 5 Material and methods

### 5.1 Animal studies

A first batch of *db/db* and heterozygote control mice *db/+* at 16 weeks of age were obtained from the Garvan Institute breeding Colonies and has been described previously [18]. A second batch of *db/db* mice (genetic background BKS(D)-*Leprdb*/JOrlRj) and their respective controls (genetic background BKS(D)-*Lepr*^*db/+*^/JorlRj) at 11 weeks of age has been obtained from Janvier Laboratories (Le Genest-Saint-Isle, France). These mice were housed in our animal facility until they reached 15 weeks of age.

For the low protein (LP) diet model, Sprague Dawley pregnant rats were fed either a LP diet (5.3 % protein, 17 % fat, 77.7 % carbohydrate) or an isocaloric control diet from day 2 of pregnancy and throughout lactation until the progeny were weaned and sacrificed at day 22.

For fasting/re-feeding cycle experiments, 12-week-old C57BL/6J female mice were divided into 3 groups. Each group of mice was fasted for 2 h and then received an oral gavage of glucose at 2 mg/g body weight (BW) to harmonize their glycemic status and glycogen stores (“fed” state). Group 1 animals (fed) were sacrificed 30 min post-gavage. Mice in group 2 (16 h fasting) were fasted for 16 h overnight, then given water orally and sacrificed 2 h later. Mice in group 3 (2 h post-prandial) were fasted for 16 h, then given an oral glucose bolus and sacrificed 2 h later. For each mouse, the success of the gavage performed after a 2 h- or 16 h fast was checked by measuring blood glucose 30 min after the *per os* administration. A blood drop was collected from a tail snip by gentle massaging and analyzed by a glucometer.

For diet-induced obese (DIO) mice studies, 8-week-old C57BL/6J male mice were fed a hypercaloric diet at 5.21 kcal/g containing 20 % protein, 20 % carbohydrate, and 60 % fat named high-fat diet (HFD) and a control diet at 3.82 kcal/g containing 20 % protein, 70 % carbohydrate, and 10 % fat for 16 weeks.

C57BL/6 male and female mice, pregnant Sprague Dawley rats as well as Wistar male rats were obtained from Janvier Laboratories (Le Genest-Saint-Isle, France).

Animal experimentation procedures are further detailed within Supplementary Material and methods.

### 5.2 Small RNA-sequencing

Small RNA-sequencing was performed on pancreatic islets isolated from 16-week-old male *db/db* mice and 22-day-old male rats exposed to protein deficiency during fetal and postnatal life versus their respective controls (see Appendix A. Supplementary Material and methods). RNA was isolated using miRNeasy micro kit and cDNA library was prepared using QIAseq miRNA NGS 48 Index IL kit. To remove tRNA-modifications that may interfere with the reverse transcription and the quantification of tRNA-derived fragments, nucleotide modifications were removed from RNA samples prior to sequencing library construction using the rtStar™ tRF&tiRNA Pretreatment kit. The kit involves deacylation of 3′-aminoacyl groups to 3′-OH for 3′ adaptor ligation, 3′-cP (2′,3′-cyclic phosphate) removal to 3′-OH for 3′ adaptor ligation, 5′-OH (hydroxyl group) phosphorylation to 5′-P for 5′ adaptor ligation, and N1-methyladenosine (m^1^A), 3-methylcytidine (m^3^C) and 1-methyl guanosine (m^1^G) demethylation.

### 5.3 tRNA-derived fragment annotation

tRNA-derived fragments (tRFs) were annotated in high-throughput RNA-sequencing data from mouse (GEO accession GSE239786), rat (GEO accession GSE239981), and human islets (GEO accession code GSE105096) [23] by computational detection using the tRNA gene algorithm tRNAscan-SE used for the genomic tRF database (GtRNAdb). The mitotRNAdb database was used for annotation of mitochondrial tRFs. Two or more reads were required to call tRFs in individual samples.

### 5.4 tRF quantification by real-time PCR

Real-time PCR quantification of tRNA-derived fragments was performed using the miRCURY LNA Universal RT microRNA PCR system starting from 160 ng of RNA. To calculate relative RNA levels between different samples, we employed the delta–delta Ct method, (2^–ΔΔCt^). The obtained tRF relative abundance value was normalized by dividing it with the mean relative expression of two microRNAs, miR-7 and let-7a-5p, used as housekeeping small non-coding RNAs as they were not modified under the conditions used in this study. The input sequences used for primer design are indicated in Table S2.

### 5.5 Mouse and rat pancreatic islet isolation

Rodent pancreatic islets were isolated by collagenase digestion followed by Histopaque density gradient and handpicking as previously described [49].

### 5.6 Fluorescence-activated cell sorting (FACS)

Dissociated islet cells from adult rats and from DIO mice were sorted by FACS based on β-cell autofluorescence, as previously described [50] and further detailed in the Supplementary Material and methods.

### 5.7 Total RNA and qPCR

Total RNA from rodent and human islet cells and rat insulinoma INS 832/13 cells, was extracted with the miRNeasy kit, treated with DNase, and reverse transcribed with an M-MLV reverse transcriptase and random primers starting from 500 ng of RNA. Quantitative PCR (qPCR) was performed using the SsoAdvanced Universal SYBR Green Supermix. For amplification and measurement of the whole host tRNA, selected qPCR products were loaded in agarose gels for electrophoresis, purified with the QIAquick gel extraction kit to check the presence of a single band (molecular size between ∼ 75 and 80 bp). The efficiency of each pair of primers was evaluated employing a corresponding standard curve. RNA levels were assessed by comparing the expression of host tRNA or mRNA genes between two samples, utilizing the 2^–ΔΔCt^ method. For normalization, we calculated the ratio of 2^–ΔΔCt^ values for the gene of interest against the reference gene Hypoxanthine Phosphoribosyltransferase (Hprt). Primer sequences are listed in Table S2.

### 5.8 Culture of INS 832/13 cells

The rat insulin-secreting cell line INS 832/13 was provided by Dr. C. Newgard (Duke University) [51]. Cells were cultured in Roswell Park Memorial Institute (RPMI) 1640 GlutaMAX medium containing 11 mM glucose and 2 mM l-glutamine and supplemented with 10 % fetal calf serum, 10 mM Hepes pH 7.4, 1 mM sodium pyruvate and 0.05 mM of β-Mercaptoethanol. INS 832/13 cells were cultured at 37 °C in a humidified atmosphere (5 % CO_2_, 95 % ambient air) and tested negative for mycoplasma contamination.

### 5.9 Dissociation and culture of rat pancreatic islets

When needed, rodent and human islets were dispersed into single cells by incubation in Ca^2+^/Mg^2+^ free phosphate-buffered saline, 3 mM EGTA, and 0.002 % trypsin for 3–4 min at 37 °C. Isolated rodent islets as well as dispersed islet cells were cultured in RPMI 1640 GlutaMAX medium containing 11 mM glucose and 2 mM l-glutamine and supplemented with 10 % fetal calf serum, 10 mM Hepes pH 7.4, 1 mM sodium pyruvate, 100 mg/mL streptomycin and 100 IU/mL penicillin.

### 5.10 Special cell culture conditions and chemical treatment of INS 832/13 cells and primary rat islet cells

Rat insulinoma INS 832/13 cells were nutrient-deprived by incubating them for 24 h in glucose-free RPMI 1640 medium supplemented with low serum (LS; 0.01 % fetal calf serum) and low d-glucose (LG; 2.8 mM) concentration versus fully complemented medium as respective vehicle. Cells were incubated for 24 h with 100 nM exendin-4 or 1 % water as respective vehicle, 50 nM leptin or 0.5 % water as respective vehicle, 100 nM estradiol or 1 % absolute ethanol as respective vehicle and 100 nM progesterone 1 % absolute ethanol as respective vehicle. INS 832/13 cells and dispersed rat islet cells were incubated for 24 h with 40 nM everolimus, a mTORC1-specific inhibitor at low dose or 0.2 % dimethylsulfoxide as respective vehicle.

### 5.11 Culture of human pancreatic islets

For *in vitro* experiments, human islets from 2 female and 6 male non-diabetic organ donors (Table S3) were provided by the Centre Européen d’Etude du Diabète (CEED) of Strasbourg University (isolation protocol authorization for scientific research #PFS12-0013) and the Diabetes Unit of the Department of Clinical and Experimental Medicine (DCEM), University of Pisa. Islets from DCEM were prepared before November 30th, 2021 with the approval of the Ethics Committee of the University of Pisa, upon written consent of donors' next-of-kin. Islets were isolated by enzymatic digestion and density-gradient purification [52] and sent in M199 medium (5.5 mmol/L of glucose) to Lausanne, Switzerland where they were dispersed [53]. Human islets cells were then cultured in Connaught's Medical Research Laboratories (CMRL) 1066 medium supplemented with 10 % fetal calf serum, 100 U/mL penicillin, and 100 μg/mL streptomycin, 2 mM l-glutamine, and 10 mM HEPES. The percentage of β-cells in human islet preparations was 55.6 ± 15.1 %, determined by insulin immunofluorescence [54]. Informed consent was previously provided by all donors. Procedures complied with relevant ethical regulations and were approved by the ethics committees of the corresponding Universities.

### 5.12 Isolation of mitochondrial fraction and measurement of mt-DNA content

Separation of mitochondrial and cytosolic fractions was performed by working quickly and by keeping everything on ice throughout the whole procedure. Mitochondria were isolated from rat and human islet cells, and rat insulinoma INS 832/13 cells as previously set up and scrupulously validated [55], with some modifications that are further detailed in the Supplementary Material and methods.

Isolation of mt-DNA and nc-DNA was performed with the QIAprep Spin Miniprep Kit as per the manufacturer's instructions. Mitochondrial DNA levels were measured following established protocols [56]. The mt-DNA gene mt–CO1 and the nuclear DNA (nc-DNA) gene Ndufv1 were amplified using qPCR with SYBR-Green detection (SsoAdvanced Universal SYBR Green Supermix).

### 5.13 Cell transfection to inhibit or overexpress tRFs

INS 832/13 cells as well as dispersed rat and human islet cells were transfected with 30 pmol single-stranded antisense oligonucleotides that correspond to the complementary sequence of the targeted tRF (Qiagen #custom power inhibitor PS desalted) or 60 pmol of mimics (IDT) or scrambled control oligonucleotides using Lipofectamine 2000. For transfection in 24-well-plates 250′000 cells were seeded in 500 μL of medium while for transfections in 6-well-plates 10^6^ cells were seeded in 2000 μL of medium. Detailed sequences of antisense oligos and mimics are provided in Table S4. Cells were then cultured for 48 h or 72 h before RNA or protein extraction, or functional assays.

### 5.14 Total RNA sequencing

Transcriptomic analysis by RNA-sequencing was performed on pancreatic islets isolated from 12-week-old male Wistar rats, transfected for 72 h with control antisense oligonucleotides or with antisense oligonucleotides against mt-tRF-Leu^TAA^ (GEO accession GSE240395). Each *N* corresponds to the islets from a single rat. Details of the procedure can be found in the Supplementary Material and methods.

### 5.15 Protein digestion and liquid chromatography-tandem mass spectrometry (LC-MS/MS)

The mass spectrometry proteomics data have been deposited to the ProteomeXchange Consortium via the PRIDE [57] partner repository with the dataset identifier PXD046117 .

Frozen pellets of transfected (72 h) dispersed islet cells of 12-week-old male Wistar rats (*N* = 5) were digested with a modified version of the iST method (named miST method [58]) that is further detailed in the Supplementary Material and methods.

### 5.16 Mass spectrometry analysis

LC-MS/MS analyses were carried out on a TIMS-TOF Pro (Bruker, Bremen, Germany) mass spectrometer interfaced through a nanospray ion source (“captive spray”) to an Ultimate 3000 RSLCnano HPLC system (Dionex) (see Appendix A. Supplementary Material and methods).

### 5.17 Oligonucleotide pull-down and mass spectrometry or qRT-PCR

To elucidate the mode of action of mt-tRF-Leu^TAA^, we conducted pull-down investigations utilizing 3′-biotinylated oligos mimicking the sequence of mt-tRF-Leu^TAA^ within insulin-secreting INS 832/13 cells (described in detail in Appendix A. Supplementary Material and methods). Subsequently, we employed mass spectrometry to identify the binding partners (ProteomeXchange identifier PXD046117) (see Appendix A. Supplementary Material and methods). To determine the specificity of anti-mt-tRF-Leu^TAA^, we performed pull-down experiments utilizing 3′-biotinylated antisense oligos designed to complement the sequence of mt-tRF-Leu^TAA^ in insulin-secreting INS 832/13 cells (described in detail in Appendix A. Supplementary Material and methods). The RNAs interacting with anti-mt-tRF-Leu^TAA^ were analyzed by qRT-PCR.

### 5.18 High-resolution respirometry

To ensure an adequate number of cells per condition, the isolated islets from two rats were pooled before being partitioned in two separate groups (antisense oligonucleotides against mt-tRF-Leu^TAA^ vs control oligos, dispersed rat islet cells transfected for 72 h). Measurements of mitochondrial O_2_ consumption and mitochondrial membrane potential Δ*Ψ*_mt_ in rat pancreatic islet cells were performed using established methods for high-resolution fluorespirometry (O2k, Oroboros Instruments: limit of detection of oxygen flux at ±1 pmol O_2_ · s^−1^ · mL^−1^ [59]. Corrections of O_2_ flux for instrumental background were based on monthly instrumental quality control tests. Experiments were performed at 37 °C with constant stirring (750 rotations per minute) using a calibrated instrument (air and zero calibration of the polarographic oxygen sensor and volume calibration of the chamber) and mitochondrial respiration medium (MiR05; 0.5 mM EGTA, 3 mM MgCl_2_, 60 mM lactobionic acid, 20 mM taurine, 10 mM KH_2_PO_4_, 20 mM HEPES, 110 mM sucrose, 1 g/L BSA; pH 7.1). Respiratory capacities were assessed at saturating O_2_ concentrations in the range of ∼190 (air saturation) to 60 μM. For data acquisition and analysis DatLab 7 software was used. The procedure is further detailed in the Supplementary Material and methods.

### 5.19 Insulin secretion and insulin ELISA

Transfected rodent and human islet cells and rat insulinoma INS 832/13 cells were first incubated at 37 °C for 60 min in a Krebs–Ringer bicarbonate buffer (KRBH) containing 25 mM HEPES, pH 7.4, 0.1 % BSA, and 2 mM glucose. Thereafter, the cells were incubated at 37 °C for 45 min in KRBH-BSA solutions with 2 mM (basal) or 10 or 20 mM (stimulatory) glucose, or with 2 mM glucose and 35 mM (stimulatory) KCl in the presence or absence of 150 μM diazoxide, or with 20 mM glucose, 10 μM forskolin (Millipore SA #344270), and 100 μM 3-isobutyl-1-methylxanthine (IBMX) (stimulatory). After incubation, supernatants were collected. The cells kept at basal glucose were harvested using acid ethanol (75 % ethanol, 0.55 % HCl), and those incubated at stimulatory conditions were lysed using Triton X-100 lysis buffer to determine insulin and protein contents, respectively. Insulin levels were measured by ELISA and cellular protein contents by Bradford assay.

### 5.20 Islet cell apoptosis assessed by an ELISA DNA fragmentation detection

DNA fragmentation was determined using an ELISA Cell Death detection kit which allows the detection of nucleosomes that are disassembled from core histones (see Appendix A. Supplementary Material and methods).

### 5.21 Islet cell apoptosis assessed by counting pyknotic cells

The fraction of apoptotic cells was assessed by incubating rat islet cells with 1 mg/mL Hoechst 33342 at 37 °C for 3 min and counting the cells displaying pycnotic nuclei under a fluorescence microscope (AxioCam MRc5, Zeiss). A 24 h incubation with the pro-inflammatory cytokines IL-1β (0.1 ng/mL), TNF-α (10 ng/mL), and IFN-γ (30 ng/mL) was used to induce cell death. A minimum of 900 cells were counted per condition.

### 5.22 Assessment of β-cell proliferation and apoptosis by immunocytochemistry

Proliferative β-cells were assessed using rat insulinoma INS 832/13 cells. Apoptotic β-cells were assessed using rat insulinoma INS 832/13 cells and dispersed human islet cells. Details of the procedure are provided in the Supplementary Material and methods.

### 5.23 Quantification and statistical analysis

Data analysis as well as graphs and plots were performed in Excel, R package DESeq2 (version 4.2.0), DatLab 7 (Oroboros Instruments) and GraphPad Prism version 8.0.0 for Windows, GraphPad Software, San Diego, California USA, www.graphpad.com. All data (unless otherwise noted) is presented as mean ± standard deviation. Statistical significance was considered whenever *p*-values were ≤0.05.

To compare a data set to a control value set to 1, one-sample Student's *t*-test was used. For pairwise comparisons, statistical differences were assessed by two-tailed paired or unpaired Student's *t*-test. In the case of multiple comparisons involving a single variable, one-way or two-way ANOVA was applied followed by the appropriate parametric or nonparametric post-hoc test.

All subsequent data processing of proteomics analyses were done with the Perseus software package (version 1.6.15.0) [60]. Perseus was used to remove contaminant proteins, log2-transformation of intensity values, data normalization and missing values imputation if needed, and statistical *t*-tests with multiple-testing correction (FDR ≤0.05). The difference of means obtained from the tests were used for 1D enrichment analysis on associated GO/KEGG annotations as described [61]. The enrichment analysis was also FDR-filtered (Benjamini-Hochberg, *q*-value ≤0.02).

We performed enrichment analysis using REACTOME terms which were considered significant at the FDR adjusted *p*-value ≤0.05 (-log10 ≤ 10), *p*-value ≤0.025 (-log10 ≤ 5), *p*-value ≤0.01 (-log10 ≤ 2). The resultant significant terms were visualized in a network layout where enriched pathways were indicated as color-coded circular nodes, with node size corresponding to enrichment *p*-value. The overlap of proteins shared within common REACTOME terms was evaluated using the Cohen's kappa coefficient, a statistical analysis that allows to assess inter-variable agreement (corrected for chance agreement) for categorical variables [62]. Kappa score was calculated as followed: k = (p_0_ – p_e_)/(1 – p_e_), where p_0_ is the relative observed agreement amongst variables and p_e_ the hypothetical probability of chance agreement. Nodes were connected using edges, with edge thickness corresponding to kappa score – wherein higher kappa values denote stronger levels of agreement. Hypergeometric distribution was adjusted with the Benjamini-Hochberg false discovery rate procedure.

## Funding

This work was supported by the 10.13039/100000001Swiss National Science Foundation (#310030_188447 and #310030_219252 to R.R. and #194964 to C.D.). M.S. and P.M. were supported by the 10.13039/501100000780European Union – Next Generation EU through the Italian Ministry of University and Research, under PNRR M4C2-I1.3, Project PE_00000019 “HEAL ITALIA”.

## CRediT authorship contribution statement

**Cecile Jacovetti:** Writing – review & editing, Writing – original draft, Methodology, Formal analysis, Data curation, Conceptualization. **Chris Donnelly:** Writing – review & editing, Writing – original draft, Methodology, Funding acquisition, Formal analysis, Data curation, Conceptualization. **Véronique Menoud:** Data curation. **Mara Suleiman:** Data curation. **Cristina Cosentino:** Data curation. **Jonathan Sobel:** Formal analysis. **Kejing Wu:** Formal analysis. **Karim Bouzakri:** Validation, Funding acquisition. **Piero Marchetti:** Validation, Funding acquisition. **Claudiane Guay:** Writing – review & editing, Methodology, Data curation. **Bengt Kayser:** Writing – review & editing, Supervision, Funding acquisition, Conceptualization. **Romano Regazzi:** Writing – review & editing, Funding acquisition, Conceptualization.

## Declaration of competing interest

We have no conflict of interest to declare.

## Data Availability

Data will be made available on request.
