# Supplementary material for: The mitochondrial tRNA-derived fragment, mt-tRF-LeuTAA, couples mitochondrial metabolism to insulin secretion
Source: Mol Metab. 2024 May 3;84:101955. doi: 10.1016/j.molmet.2024.101955 (PMC11112368; doi:10.1016/j.molmet.2024.101955)
Supplement: Multimedia component 1 [file mmc1.pdf]

A

db/db mice (16-wk-old)	Body weight (g)	Glycemia (mmol/L)	Assays performed
*db/+*_1	25.7	7.7	qRT-PCR on pancreatic islets
*db/+*_2	27.8	7.5	Small RNA-seq & qRT-PCR on pancreatic islets
*db/+*_3	28.1	7.5	
*db/+*_4	23.6	8	Small RNA-seq on pancreatic islets
*db/db*_2	42.3	14.5	Small RNA-seq & qRT-PCR on pancreatic islets
*db/db*_3	49.9	17.1	
*db/db*_4	51.8	19	

db/db mice (15-wk-old)	Body weight (g)	Glycemia (mmol/L)	Assays performed
*db/+*_a	22.5	5.8	qRT-PCR on pancreatic islets, Gastricnemius muscle, liver and WAT
*db/+*_b	22.6	4.7	
*db/+*_c	23.9	5	
*db/db*_a	41.5	20.8	qRT-PCR on pancreatic islets, Gastricnemius muscle, liver and WAT
*db/db*_b	53.6	24.6	
*db/db*_c	53.8	21.7	
*db/db*_d	53	25.6	

db/db mice (14-wk-old)	Body weight (g)	Glycemia (mmol/L)	Assays performed
*db/+*_A	21.9	4.1	mt-DNA measurement on pancreatic islets
*db/+*_B	20	5.4	
*db/+*_C	18.7	5.8	
*db/db*_A	37.2	16.2	mt-DNA measurement on pancreatic islets
*db/db*_B	39.6	18.9	
*db/db*_C	52.5	24.1	
*db/db*_D	42.7	22.2	

B

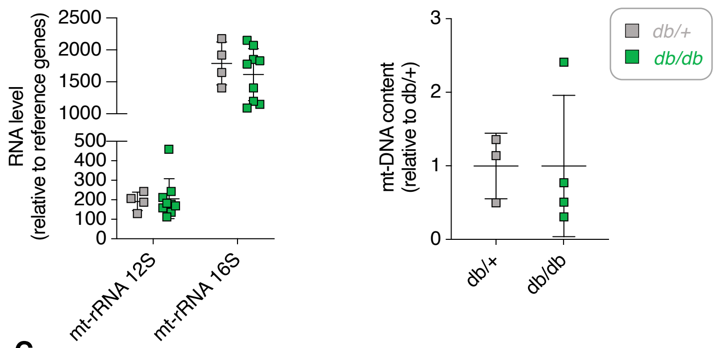

C

Rats (22-day-old)	Body weight (g)	Glycemia (mmol/L)	Assays performed
CD-exposed_1	54.5	4.6	Small RNA-seq & qRT-PCR on pancreatic islets
CD-exposed_2	55.3	3.9	
CD-exposed_3	54.2	3.8	
CD-exposed_4	53	4.1	RNA-seq on pancreatic islets
CD-exposed_5	49	3.1	
LP-exposed_1	19.4	3.2	Small RNA-seq & qRT-PCR on pancreatic islets
LP-exposed_2	19.8	4.1	
LP-exposed_3	17.8	3.9	
LP-exposed_4	17.9	5	

D

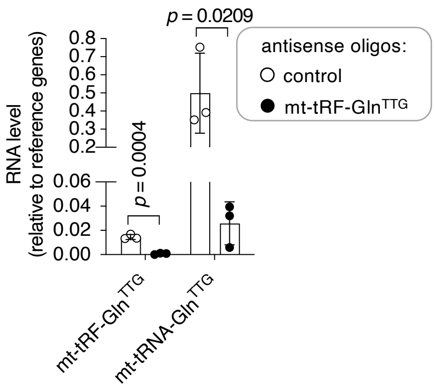

E

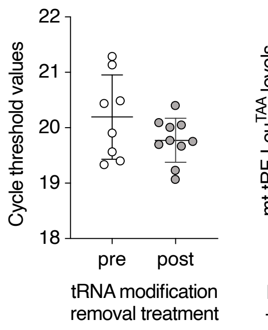

F

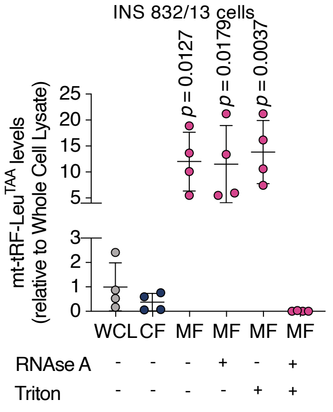

A

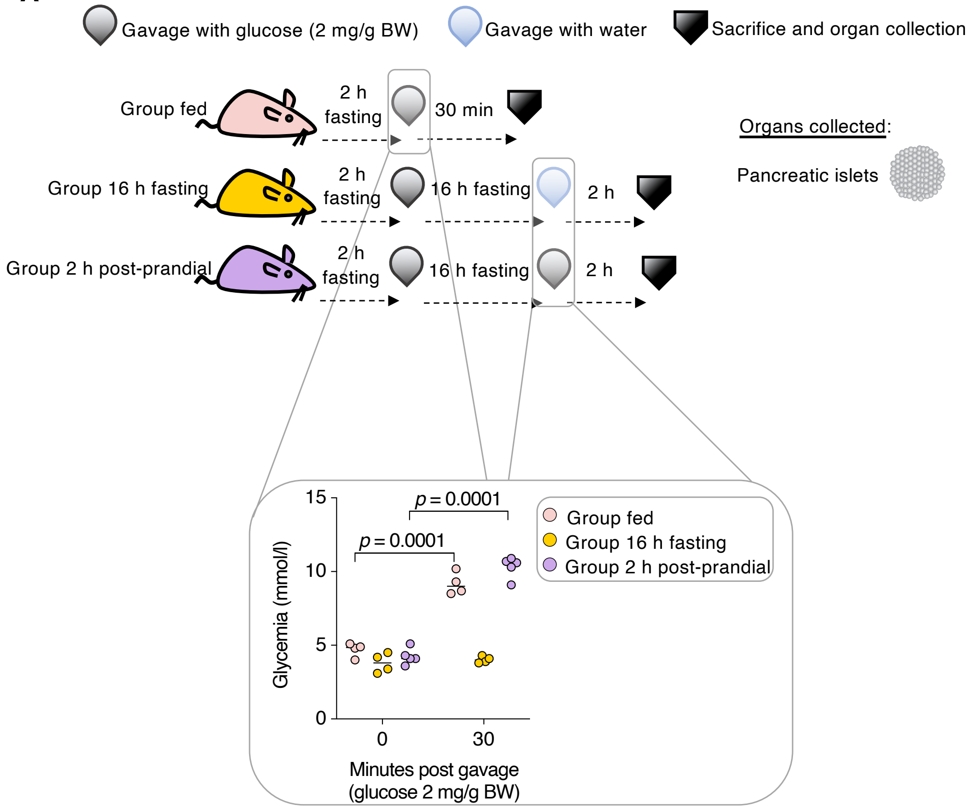

B

DIO mice (HFD for 16 weeks)	Body weight (g)	Glycemia (mmol/L)	Assays performed
Lean_1	29.3	5.9	qRT-PCR on FAC-sorted ß-cells and muscle
Lean_2	30	7.7	
Lean_3	29	7.6	
Lean_4	28.3	8.2	
Lean_5	29.2	6.8	qRT-PCR on muscle
Lean_6	30.3	5.5	
Lean_7	30.8	6.4	
Lean_8	29.6	7.7	
Lean_9	31.7	7.5	
DIO_1	39.9	5.1	qRT-PCR on FAC-sorted ß-cells and muscle
DIO_2	39.9	7.3	
DIO_3	36.6	5.4	
DIO_4	38.9	7.2	
DIO_5	40.5	7.2	
DIO_6	38.3	7.2	
DIO_7	43.7	6.5	qRT-PCR on muscle
DIO_8	36.4	7.8	

DIO mice (HFD for 17 weeks)	Body weight (g)	Glycemia (mmol/L)	Assays performed
Lean_1	30	7.1	qRT-PCR on liver and eWAT
Lean_2	29.4	7.5	
Lean_3	31.2	5.9	
Lean_4	26.4	6	
DIO_1	38.5	6.8	qRT-PCR on liver and eWAT
DIO_2	46.1	6.2	
DIO_3	36.9	7	
DIO_4	41.7	6.1	

A

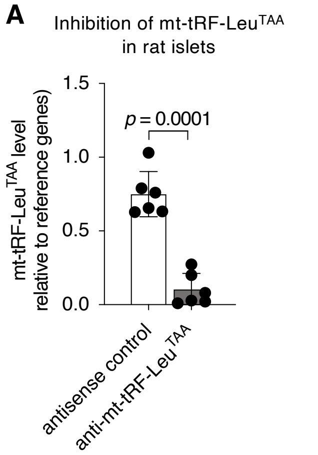

B

Transcriptomics	Proteomics
13 733 protein-coding genes detected	7145 proteins detected
**2412 genes are significantly up-regulated** (adjusted $p \leq 0.05$) amongst which **906 genes are significantly up-regulated ≥ 2 FC**	**321 proteins are significantly up-regulated** (adjusted $p \leq 0.05$)
**2431 genes are significantly down-regulated** (adjusted $p \leq 0.05$) amongst which **31 genes are significantly down-regulated ≥ 2 FC**	**321 proteins are significantly downregulated** (adjusted $p \leq 0.05$)
**Amongst the 4843 genes that are significantly changed:**<br>mt-DNA encoded transcripts are not affected	**Amongst the 642 proteins that are significantly changed:**<br>3 are mitochondrially-encoded proteins (mt-ATP8, mt-CO1 and mt-CO3)<br>91 are nuclear-encoded proteins that are imported into the mitochondria (FC ≥ 2, adjusted $p \leq 0.05$)

C

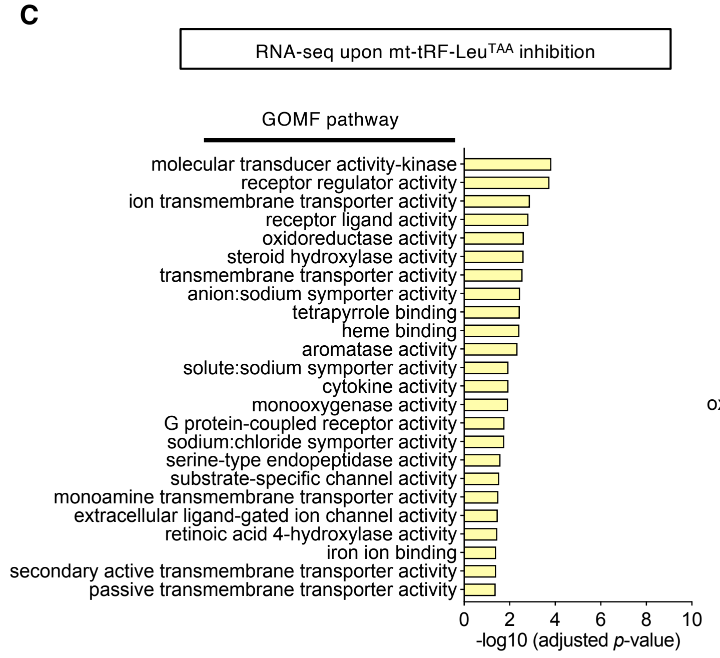

D

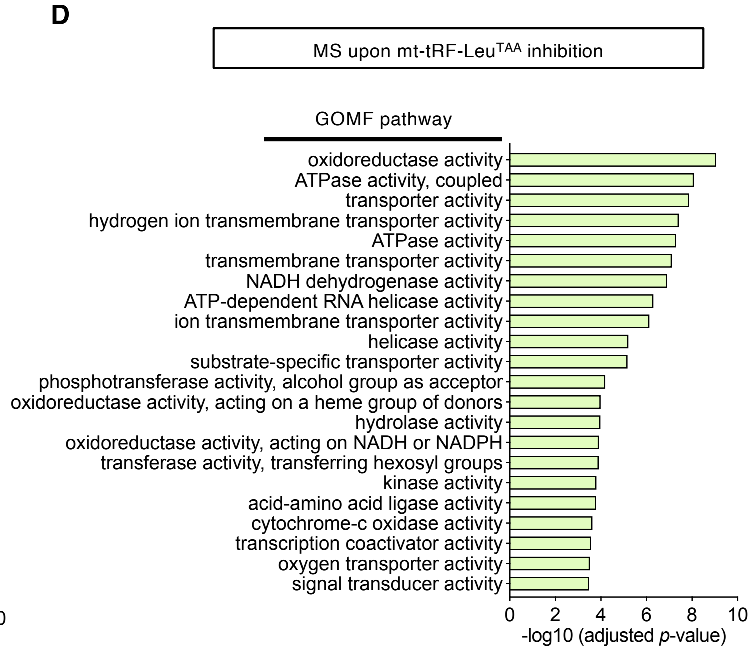

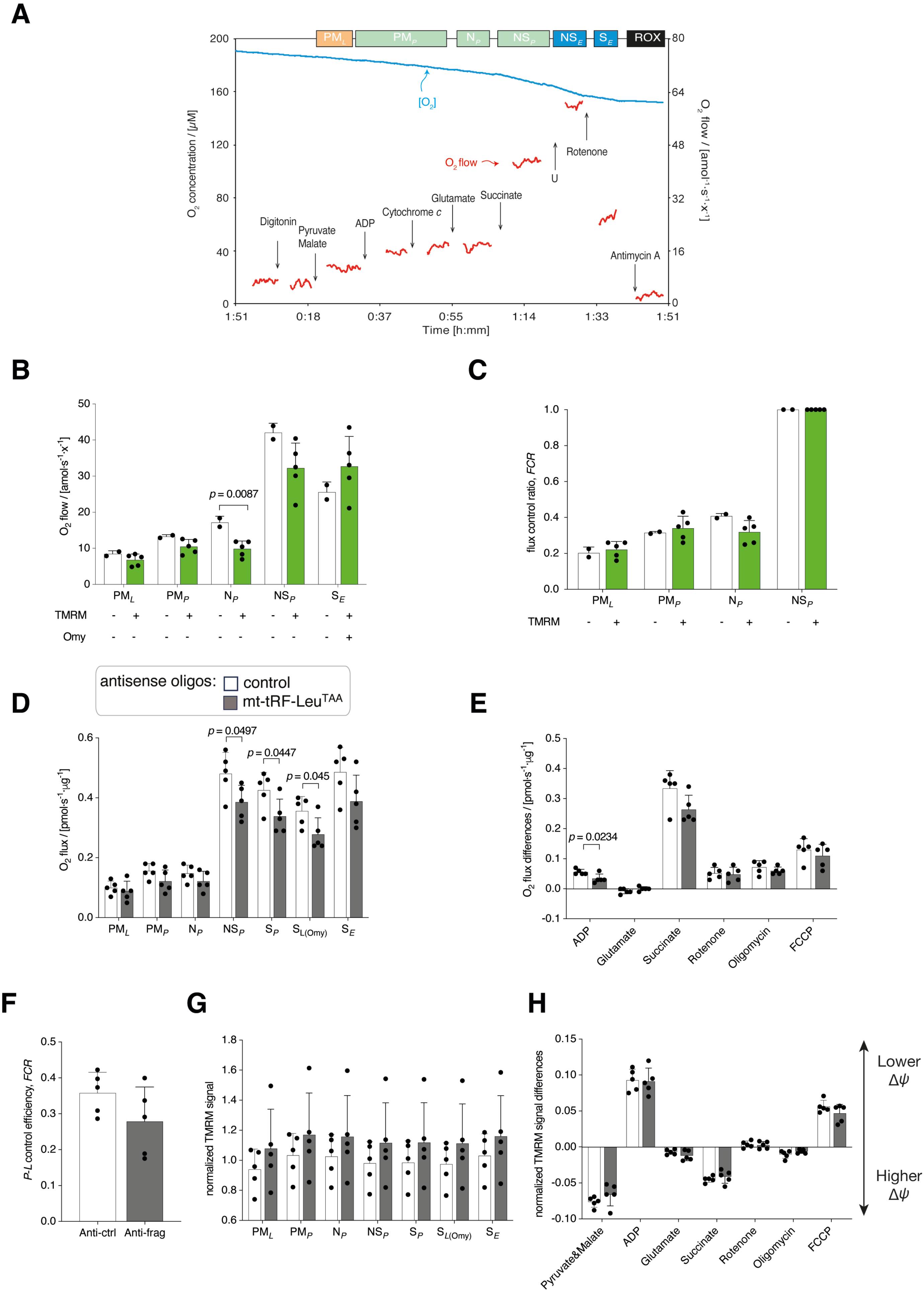
A
PM$_L$
PM$_P$
N$_P$
NS$_P$
NS$_E$
S$_E$
ROX
O$_2$ concentration / [μM]
O$_2$ flow / [amol^{-1}·s^{-1}·x^{-1}]
[O$_2$]
O$_2$ flow
Digitonin
Pyruvate Malate
ADP
Cytochrome c
Glutamate
Succinate
U
Rotenone
Antimycin A
Time [h:mm]
B
C
flux control ratio, FCR
TMRM
Omy
p = 0.0087
antisense oligos: control
mt-tRF-Leu^{TAA}
D
O$_2$ flux / [pmol·s^{-1}·μg^{-1}]
p = 0.0497
p = 0.0447
p = 0.045
S$_{L(Omy)}$
E
O$_2$ flux differences / [pmol·s^{-1}·μg^{-1}]
p = 0.0234
ADP
Glutamate
Succinate
Rotenone
Oligomycin
FCCP
F
P-L control efficiency, FCR
Anti-ctrl
Anti-frag
G
normalized TMRM signal
H
normalized TMRM signal differences
Pyruvate&Malate
Lower Δψ
Higher Δψ

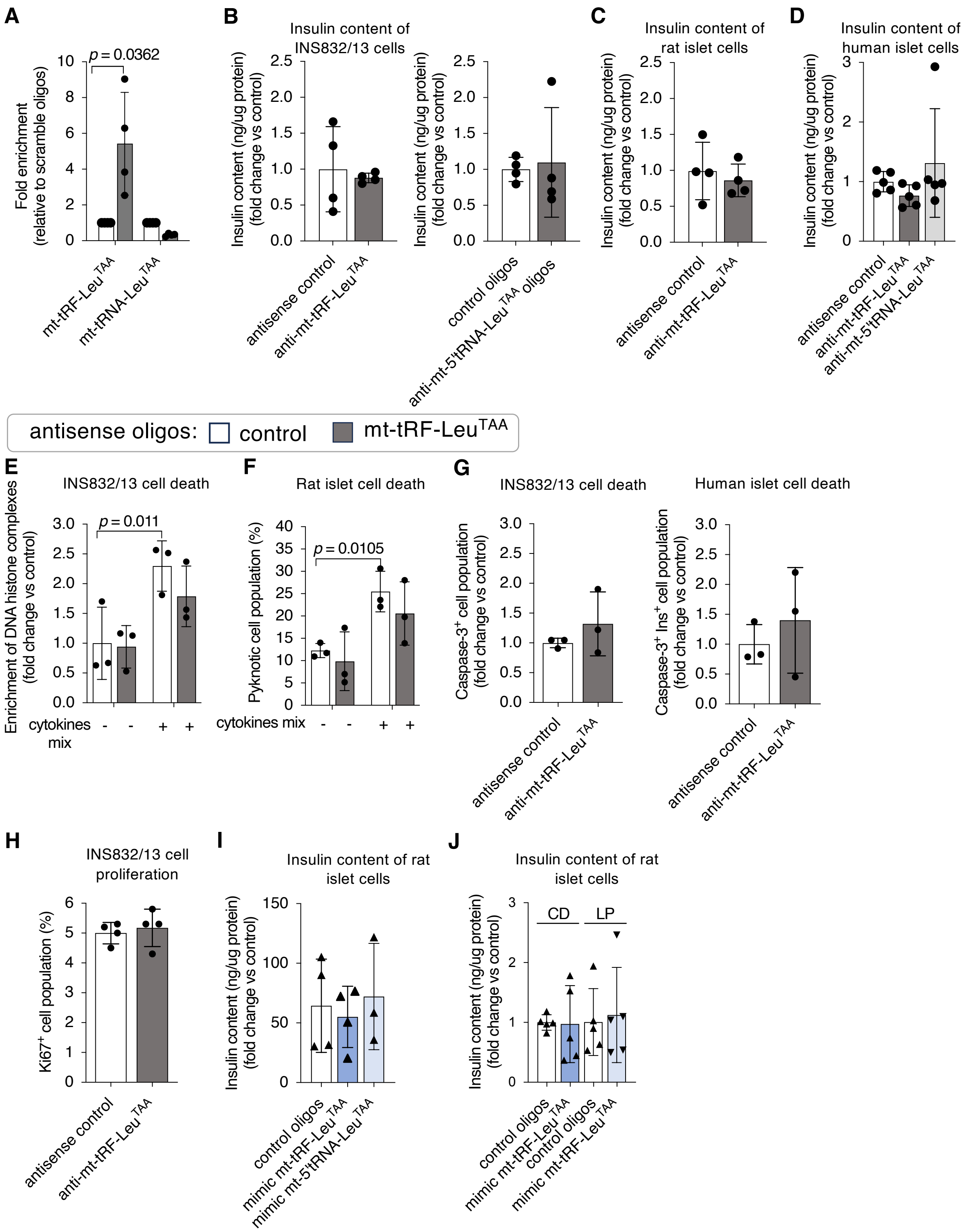

A
p = 0.0362
Fold enrichment (relative to scramble oligos)
mt-tRF-Leu TAA
mt-tRNA-Leu TAA
B
Insulin content of INS832/13 cells
Insulin content (ng/ug protein) (fold change vs control)
antisense control
anti-mt-tRF-Leu TAA
control oligos
anti-mt-5'tRNA-Leu TAA oligos
C
Insulin content of rat islet cells
D
Insulin content of human islet cells
anti-mt-5'tRNA-Leu TAA
antisense oligos: control mt-tRF-Leu TAA
E
INS832/13 cell death
p = 0.011
Enrichment of DNA histone complexes (fold change vs control)
cytokines mix
F
Rat islet cell death
p = 0.0105
Pyknotic cell population (%)
G
INS832/13 cell death
Human islet cell death
Caspase-3+ cell population (fold change vs control)
Caspase-3+ Ins+ cell population (fold change vs control)
H
INS832/13 cell proliferation
Ki67+ cell population (%)
I
Insulin content of rat islet cells
mimic mt-tRF-Leu TAA
mimic mt-5'tRNA-Leu TAA
J
CD
LP

Supplementary Table 1. List of the proteins pulled-down with biotinylated mt-tRF-Leu^{TAA}.

Display of the protein and gene names with their corresponding enrichment, t-test followed by Benjamini-Hochberg method, fold change cutoff ≥ 6, adjusted *p*-value ≤ 0.05.

Protein name	Gene name	Fold enrichment	q-value
Peptidyl-prolyl cis-trans isomerase D = Cyclophilin D	Ppid	159.7	4.79E-03
Actin-related protein 2/3 complex subunit 2	Arpc2	69.5	1.12E-02
Poly(rC) binding protein 2	Pcbp2	60.7	1.47E-03
Poly(rC) binding protein 3	Pcbp3	53.9	1.66E-03
Nuclear migration protein nudC	Nudc	44.6	7.74E-03
Succinyl-CoA ligase subunit beta	Suclg2	29.6	1.10E-02
Dihydrofolate reductase	Dhfr	24.1	2.81E-02
Nucleoside diphosphate kinase	NDK3	16.4	8.99E-03
60S ribosomal protein L29	Rpl29	14.7	1.66E-03
Golgi resident protein GCP60	Acbd3	14.5	6.88E-03
Endothelial differentiation-related factor 1	Edf1	11.3	1.32E-02
Succinate dehydrogenase [ubiquinone] flavoprotein subunit	Sdha	10.2	4.90E-02
Developmentally regulated GTP binding protein 1	Drg1	9.8	4.52E-03
Syntenin-1	Sdcbp	9.6	5.45E-03
Coiled-coil domain-containing protein 47	Ccdc47	9.6	2.62E-02
Secretogranin-3	Scg3	9.3	1.47E-02
Platelet-activating factor acetylhydrolase IB subunit gamma	Pafah1b3	9.1	4.74E-03
Mitochondrial import receptor subunit TOM20 homolog	Tomm20	9.1	2.53E-03
Leucine-rich PPR motif-containing protein	Lrpprc	8.5	3.21E-02
DNA polymerase delta interacting protein 2	Poldip2	8.5	5.60E-03
Dipeptidyl peptidase 2	Dpp7	7.3	2.95E-02
NADH dehydrogenase [ubiquinone] 1 alpha subcomplex subunit 12	Ndufa12	6.8	1.66E-03
Mal, T cell differentiation protein 2	Mal2	6.0	5.60E-03
AP-2 complex subunit alpha-2	Ap2a2	6.0	1.38E-02

Supplementary Table 2. List of input sequences used for primer design and list of primer sequences.

Input sequences used for primer design using the miRCURY LNA Universal RT microRNA PCR system (Qiagen)		
Species	Targets	tRF sequences
Rattus norvegicus	mt-tRF-Leu^{TAA}	AAGACTTAAAACCTTGTTCCCAGAGGTTCAAATCCTCT
	mt-5'tRNA-Leu^{TAA}	ATTAGGGTGGCAGAGCCAGGTAATTGCGT
	mt-tRF-Gln^{TTG}	TAGGATAGGGTGTATTGGTAGCACGGAGAATTTTGAATTCTTAG GTGTAGG
	nc-tRF-Asp^{GTC}	ACGCGGGAGACCGGGGTTCGATTCCCCGACGGGGAG
Mus musculus	mt-tRF-Leu^{TAA}	TGCGTAAGACTTAAAACCTTGTTCCCAGAGGTTCAAATCCTCTC C
Homo sapiens	mt-tRF-Leu^{TAA}	TCGCATAAAACTTAAAACTTTACAGTCAGAGGTTCAATT

Primers used with SsoAdvanced Universal SYBR Green Supermix (Biorad)			
Species	Targets	Forward primers (5'-3')	Reverse primers (5'-3')
Rattus norvegicus	host-mt-tRNA-Leu^{TAA}	AGGGTGGCAGAGCCAGGTAA	GTATTAGGGAGAGGATTT GA
	mt-CO1	CTAGCCGGAAATCTAGCCCA	TGCGGCTAGTACTGGTAG TG
	mt-rRNA 12S	CAAACTGGGATTAGATACCCCA CTAT	GAGGGTGACGGGCGGTGT GT
	mt-rRNA 16S	ACCGCAAGGGAAAGATGAAA	GCCACATAGACGAGTTGA TTC
	Ndufv1	CGGGTATCTGTGCGTTTCAG	TGTCTTGTACCAGTCACC CC
Mus musculus	host-mt-tRNA-Leu^{TAA}	ATTAGGGTGGCAGAGCCAG	AACAAGGTTTTAAGTCTT A
Rattus norvegicus and Mus musculus	Hprt	AGTCCCAGCGTCGTGATTAG	AATCCAGCAGGTCAGCAA AG

Supplementary Table 3. Detailed information regarding the non-diabetic organ donors.

Sample	Age (years)	Gender	BMI (kg/m^2)	Source of islets
1	62	female	23.5	DCEM Italy
2	65	male	27.8	DCEM Italy
3	64	male	22.7	DCEM Italy
4	86	male	26.1	DCEM Italy
5	81	male	22.9	DCEM Italy
6	84	female	23.7	DCEM Italy
7	71	male	34.1	CEED France
8	66	male	29.4	CEED France

Supplementary Table 4. Sequences of antisense oligos and mimics.

Species	Oligonucleotide types	Name and sequence of the tRF targeted	Name and sequence of the oligonucleotides
Rattus norvegicus	Antisense	Rno-mt-tRF-Leu^{TAA}<br>AAGACTTAAAACCTTGTTCCCAGAGGTTCAAATCCTCT	Anti-rno-mt-tRF-Leu^{TAA}<br>TGGGAACAAGGTTTTAAGTCT
		Rno-mt-5'tRF-Leu^{TAA}<br>ATTAGGGTGGCAGAGCCAGGTAATTGCGT	Anti-rno-mt-5'tRF-Leu^{TAA}<br>AATTACCTGGCTCTGCCACC
		Rno-mt-tRF-Gln^{TTG}<br>TTTTGAATTCTTAGGTGTAGGTTCAATTCCTATTGTC	Anti-rno-mt-tRF-Gln^{TTG}<br>TGAACCTACACCTAAGAATTCAAA
	Mimic	Rno-mt-tRF-Leu^{TAA}<br>AAGACTTAAAACCTTGTTCCCAGAGGTTCAAATCCTCT	Rno-mt-tRF-Leu^{TAA} mimic<br>AAGACTTAAAACCTTGTTCCCAGAGGTTCAAATCCTCT
		Rno-mt-5'tRF-Leu^{TAA}<br>ATTAGGGTGGCAGAGCCAGGTAATTGCGT	Rno-mt-5'tRF-Leu^{TAA} mimic<br>ATTAGGGTGGCAGAGCCAGGTAATTGCGT
Mus musculus	Antisense	Mmu-mt-tRF-Leu^{TAA}<br>TGCGTAAGACTTAAAACCTTGTTCCCAGAGGTTCAAATCCTCTCC	Anti-mmu-mt-tRF-Leu^{TAA}<br>TGGGAACAAGGTTTTAAGTCT
Homo sapiens	Antisense	Hsa-mt-tRF-Leu^{TAA}<br>TCGCATAAAACTTAAAACTTTACAGTCAGAGGTTCAATT	Anti-hsa-mt-tRF-Leu^{TAA}<br>AATTGAACCTCTGACTGT
		Hsa-5'tRF-Leu^{TAA}<br>GTTAAGATGGCAGAGCCCGGTAATCGCATAAAACTTAAAACTTTAC	Anti-hsa-mt-5'tRF-Leu^{TAA}<br>AGTTTTAAGTTTTATGCGATT
